# Cryo-EM structure of Shiga toxin 2 in complex with the native ribosomal P-stalk reveals residues involved in the binding interaction

**DOI:** 10.1016/j.jbc.2022.102795

**Published:** 2022-12-15

**Authors:** Arkadiusz W. Kulczyk, Carlos Oscar S. Sorzano, Przemysław Grela, Marek Tchórzewski, Nilgun E. Tumer, Xiao-Ping Li

**Affiliations:** 1 Institute for Quantitative Biomedicine, Department of Biochemistry and Microbiology, Rutgers University, Piscataway, New Jersey, USA; 2 Biocomputing Unit, Centro Nacional de Biotecnología (CNB), Consejo Superior de Investigaciones Científicas (CSIC), Madrid, Spain; 3 Department of Molecular Biology, Institute of Biological Sciences, Maria Curie-Skłodowska University, Lublin, Poland; 4 Department of Plant Biology, School of Environmental and Biological Sciences, Rutgers University, New Brunswick, New Jersey, USA

**Keywords:** Shiga toxin, ribosomal-inactivating protein, ribosomal P-stalk, cryo-EM, CTD, C-terminal domain, CTF, contrast transfer function, ER, endoplasmic reticulum, HUS, hemolytic uremic syndrome, IDP, intrinsically disordered protein, NTD, N-terminal domain, PCA, principal component analysis, PDB, Protein Data Bank, RIP, ribosome-inactivating protein, RTA, ricin toxin A, SPR, surface plasmon resonance, SRL, sarcin–ricin loop, STEC, Shiga toxin producing *Escherichia coli*, Stx2a, Shiga toxin 2a, Stx2A, A subunit of Stx2a, TCS, trichosanthin, trGTPase, translational GTPase

## Abstract

Shiga toxin 2a (Stx2a) is the virulence factor of enterohemorrhagic *Escherichia coli.* The catalytic A1 subunit of Stx2a (Stx2A1) interacts with the ribosomal P-stalk for loading onto the ribosome and depurination of the sarcin–ricin loop, which halts protein synthesis. Because of the intrinsic flexibility of the P-stalk, a structure of the Stx2a–P-stalk complex is currently unknown. We demonstrated that the native P-stalk pentamer binds to Stx2a with nanomolar affinity, and we employed cryo-EM to determine a structure of the 72 kDa Stx2a complexed with the P-stalk. The structure identifies Stx2A1 residues involved in binding and reveals that Stx2a is anchored to the P-stalk *via* only the last six amino acids from the C-terminal domain of a single P-protein. For the first time, the cryo-EM structure shows the loop connecting Stx2A1 and Stx2A2, which is critical for activation of the toxin. Our principal component analysis of the cryo-EM data reveals the intrinsic dynamics of the Stx2a–P-stalk interaction, including conformational changes in the P-stalk binding site occurring upon complex formation. Our computational analysis unveils the propensity for structural rearrangements within the C-terminal domain, with its C-terminal six amino acids transitioning from a random coil to an α-helix upon binding to Stx2a. In conclusion, our cryo-EM structure sheds new light into the dynamics of the Stx2a–P-stalk interaction and indicates that the binding interface between Stx2a and the P-stalk is the potential target for drug discovery.

Shiga toxin (Stx) producing *Escherichia coli* (STEC), such as *E. coli* O157:H7 and *Shigella dysenteriae* are emerging foodborne pathogens, which cause severe morbidity and mortality among millions of people worldwide (1, 2). The recent outbreaks of STEC infections in the United States and the emergence of highly virulent and antibiotic-resistant strains highlight the importance of these pathogens for food safety and public health (3, 4). STEC infections can result in gastrointestinal disease such as hemorrhagic colitis and may progress to hemolytic uremic syndrome (HUS) (1). There are no therapeutics against STEC infections. Stxs are the virulence factors of STEC, and their release is critical for the development of HUS. There are two main groups of Stx toxins, Stx1a and Stx2a, both encoded by lysogenic lambdoid phages (5). Although Stx1a and Stx2a are similar in structure and function, Stx2a is more often associated with progression to severe disease such as HUS (2).

Stx2a holotoxin is a ribosome-inactivating protein (RIP), which belongs to a family of AB_5_ toxins, consisting of a monomeric A subunit (Stx2A) and a pentameric B subunit (Stx2B). Stx2B binds to globotriaosylceramide (Gb3) located on the surface of target cells. After binding, the holotoxin is internalized by clathrin-mediated endocytosis and traffics to the Golgi apparatus, where Stx2A is cleaved into catalytically active A1 (Stx2A1) and nonactive A2 (Stx2A2) fragments, at the furin-sensitive loop connecting the two fragments (6, 7). After the proteolytic cleavage, the structure of Stx2a remains intact because of a disulfide bond connecting Stx2A1 and Stx2A2 and the binding of Stx2A2 to Stx2B. The holotoxin is then transported to the endoplasmic reticulum (ER), where the disulfide bond is reduced, leading to dissociation of Stx2A1 from Stx2A2 to Stx2B. Subsequently, Stx2A1 undergoes retrotranslocation from the ER to the cytosol, where it associates with the ribosomal P-stalk.

The ribosomal P-stalk is the main landing platform for RIPs on the eukaryotic ribosome. The P-stalk is composed of five P-proteins, namely uL10 protein (former name P0) (8) forming the base of the stalk, and two P1–P2 heterodimers, each binding to uL10 (9, 10). The P-stalk confers extraordinary specificity for the RIPs toward the eukaryotic ribosome (11). Each P-protein consists of a structured N-terminal domain (NTD) and an unstructured C-terminal domain (CTD). Because of the intrinsic flexibility of the ribosomal P-stalk, particularly in the CTDs of the ribosomal P-proteins, its structure has not yet been solved (12, 13, 14). Stx2A1 (15, 16) as well as other RIPs (11, 17) bind to the conserved CTDs (Fig. 1), with a prominent role of the CTDs from the P1 proteins (18). This interaction triggers a conformational change in the CTD, which loads Stx2A1 onto the sarcin–ricin loop (SRL) of 28S rRNA in the large ribosomal subunit (Fig. 1). The binding of Stx2A1 to the SRL is mediated *via* its *N*-glycosidase active site. The active and the P-stalk-binding sites are located on the opposite surfaces of Stx2A1 (Fig. 1). Stx2A1 catalyzes the hydrolysis of the *N*-glycosidic bond in the conserved SRL adenine 2660/3027 (*E. coli*/*Saccharomyces cerevisiae*) causing its depurination. Mechanistic outcome of depurination is distortion of the conformation of SRL, which in turn affects the binding and activity of translational GTPases (trGTPases), subsequently leading to inhibition of the elongation step in protein synthesis (19). While interactions of RIPs with short polypeptides mimicking the CTD of the P-proteins have been characterized biochemically and structurally (14, 16), and a structure of Stx2a bound to the 11 amino-acid peptide (P11) was determined by X-ray crystallography (20), a structure of any RIP complexed with its physiologically relevant partner, the P-stalk pentamer, has not yet been investigated.

**Figure 1:**
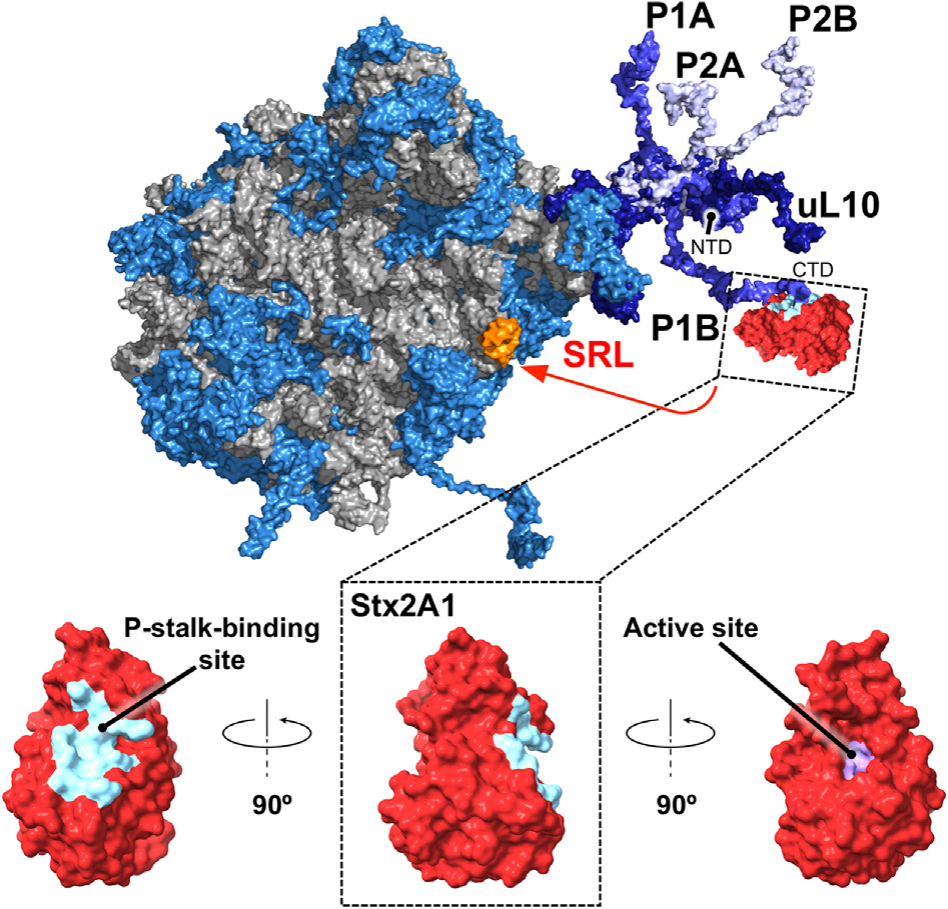
**Stx2A1 loading onto the ribosome.** A model depicting recruitment of Stx2A1 onto the sarcin–ricin loop (SRL) of 28S RNA in the large ribosomal subunit by the pentameric ribosomal P-stalk composed of two P1–P2 heterodimers bound to uL10 protein. The P-stalk proteins are labeled in the figure. *Saccharomyces cerevisiae* 26S rRNA (Protein Data Bank [PDB] ID: 3U5H) and 60S ribosomal subunit (PDB ID: 3U5I) are displayed in *gray*, ribosomal proteins are depicted in *light blue*, uL10 shown in *dark blue* is complexed with two copies of P1 proteins (P1A and P1B) and two copies of P2 proteins (P2A and P2B) (PDB ID: 4BEH) displayed in *slate* and *blue*, respectively. The N- and C-terminal domains (NTD and CTD, respectively) are labeled in one of the P1 proteins. Three different orientations of Stx2A1 (PDB ID: 2GA4) are shown at the bottom of the picture as a space-filling model in *red*. The P-stalk–binding site (*cyan*) and an active site (*purple*) are located on the opposite surfaces of Stx2A1. In the picture, the CTD of a P1B binds to the P-stalk–binding site on Stx2A1 and loads the toxin onto the SRL of 28S RNA, as indicated by the *red arrow*. The SRL is shown in *orange*. Stx2A1, A1 subunit of Stx2a.

In the current study, we employed cryo-EM and biophysical methods to examine the interaction between Stx2a and the P-stalk pentamer. Recent advances in cryo-EM provide unprecedented insights into structures of macromolecular complexes; however, structure determination of sub-100 kDa biomolecules continues to present considerable difficulties (21, 22). Here, the state-of-the-art instrumentation and multiple software processing platforms (23, 24) were used to determine a 4.1 Å cryo-EM structure of the 72 kDa Stx2a in complex with the ribosomal P-stalk, with local resolutions in the range of 3.5 to 7.5 Å. The structure shows that only a short six amino-acid fragment from the CTD of a single P-protein is involved in complex formation. In addition, the structure reveals a number of novel features including the so-far unidentified Stx2A1 residues involved in binding to the CTD and a flexible loop connecting the Stx2A1 and Stx2A2 fragments. For the first time, we show that the Stx2a–P-stalk interaction is intrinsically dynamic, with conformational changes occurring in the P-stalk-binding site of Stx2a upon complex formation. The cryo-EM structure of Stx2a–P-stalk is the first structure of any RIP in complex with the native ribosomal P-stalk. The structure provides insights into the dynamic nature of the Stx2a–P-stalk interaction and indicates that the Stx2a–P-stalk interface is a new potential target for drug discovery.

## Results

### The interaction of Stx2a with the natively assembled ribosomal P-stalk

**Figure 2:**
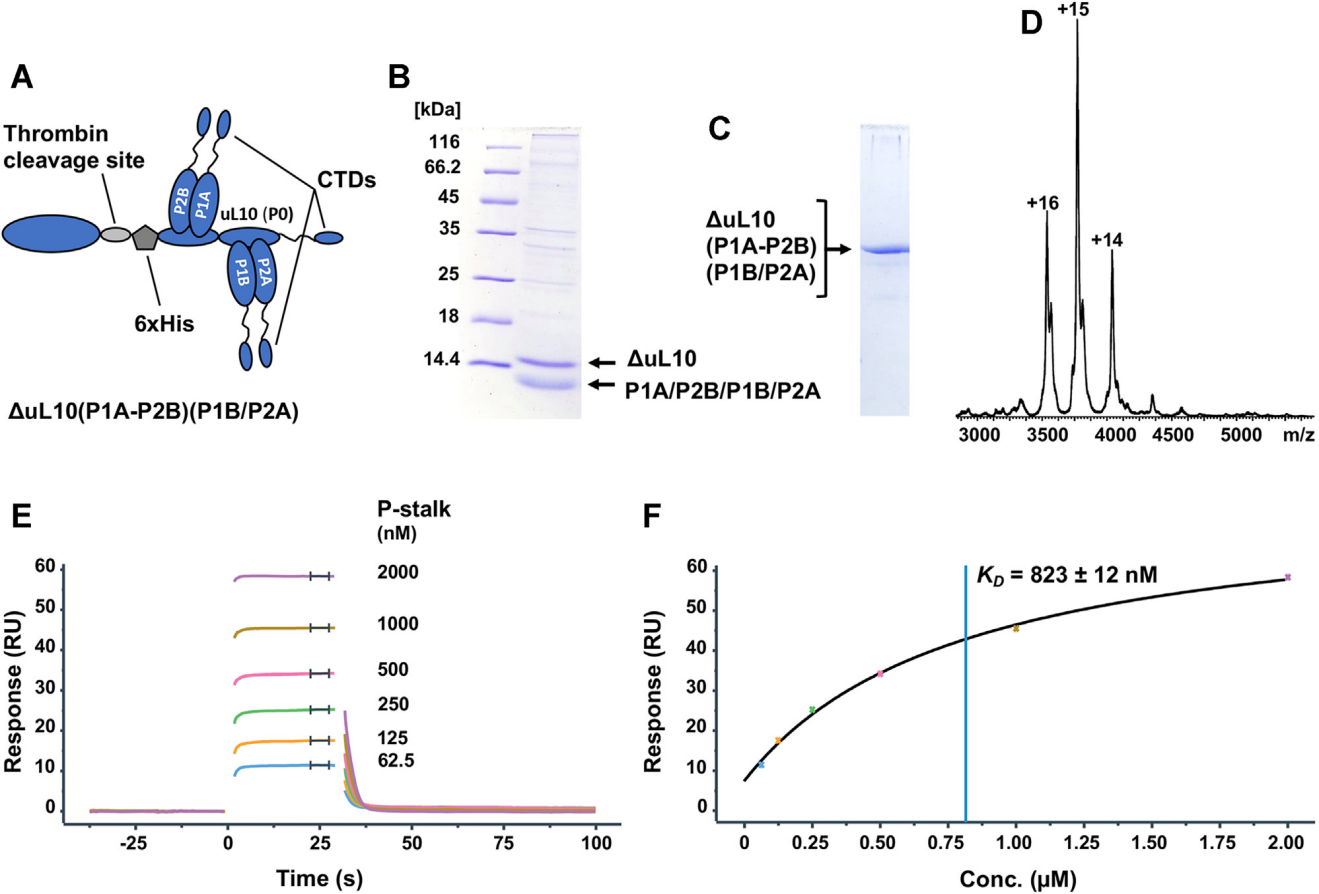
**Characterization and binding of the native ribosomal P-stalk from *Saccharomyces cerevisiae* to Stx2a.***A*, schematic representation of a genetically altered P-stalk used in the study. *B* and *C*, the purity and homogeneity of the P-stalk was assessed by SDS-PAGE and native PAGE, respectively. Low–molecular-weight bands on the denaturing gel represent individual P-proteins bound together in different combinations. *D*, native mass spectrum of the P-stalk shows three ionization states of the intact complex with a molecular weight of 56,557.73 ± 9.23. *E*, Biacore sensorgrams obtained for the P-stalk–Stx2a binding. The interaction was measured using Biacore 8K^+^. Stx2a holotoxin was immobilized on Fc2 of a CM5 chip (800–820 RU) using amine coupling. The P-stalk was flown over the surface of the chip at different concentrations listed in the figure. *F*, the *K*_*D*_ of 823 ± 12 nM (*R*_max_ = 63 ± 1 RU, offset = 7 ± 0.7 RU, Chi^2^ = 1 ± 0.1 RU^2^) for the P-stalk–Stx2a binding was calculated by fitting sensorgrams recorded in four different channels at steady state. The *blue line* denotes the *K*_*D*_. The figure shows one of the four replicates used in calculations. Stx2a, Shiga toxin 2a.

We employed surface plasmon resonance (SPR) to examine the interaction of Stx2a with the native ribosomal P-stalk isolated from *S. cerevisiae*. The pentameric P-stalk complex was obtained from the genetically engineered yeast strain, in which the gene for uL10 was modified such that the thrombin cleavage site and the 6xHis tag were introduced into the uL10 protruding domain (Fig. 2*A*), thus enabling the release of the natively assembled P-stalk from the ribosome by thrombin treatment (25). The P-stalk complex was purified to homogeneity (Fig. 2*B*). The purified P-stalk had a stable pentameric organization, as evidenced by native PAGE (Fig. 2*C*) and native mass spectrometry (Fig. 2*D*), with a molecular mass of 56.5 kDa.

To examine the interaction of Stx2a with the P-stalk, we immobilized Stx2a on the surface of the SPR chip and injected the P-stalk pentamer at the concentration ranging from 62.5 to 2 μM (Fig. 2*E*). The *K*_*D*_ obtained by fitting the binding curves at steady state is 823 nM (Fig. 2*F*). The interaction displays fast on/off kinetics, the fact confirmed by binding measurements performed using a single-cycle injection method (Fig. S1*A*). We also measured the binding between Stx2a and P11 (Fig. S2*B*). The P11 was previously cocrystallized with Stx2a (20). The *K*_*D*_ for Stx2a–P11 interaction is 48 μM (Fig. S2*C*). Thus, Stx2a binds to the P-stalk pentamer with nearly 60-fold higher affinity than it does to P11, demonstrating that the native P-stalk represents the optimal binding partner for Stx2a.

### The cryo-EM structure of the Stx2a–ribosomal P-stalk complex

**Figure 3:**
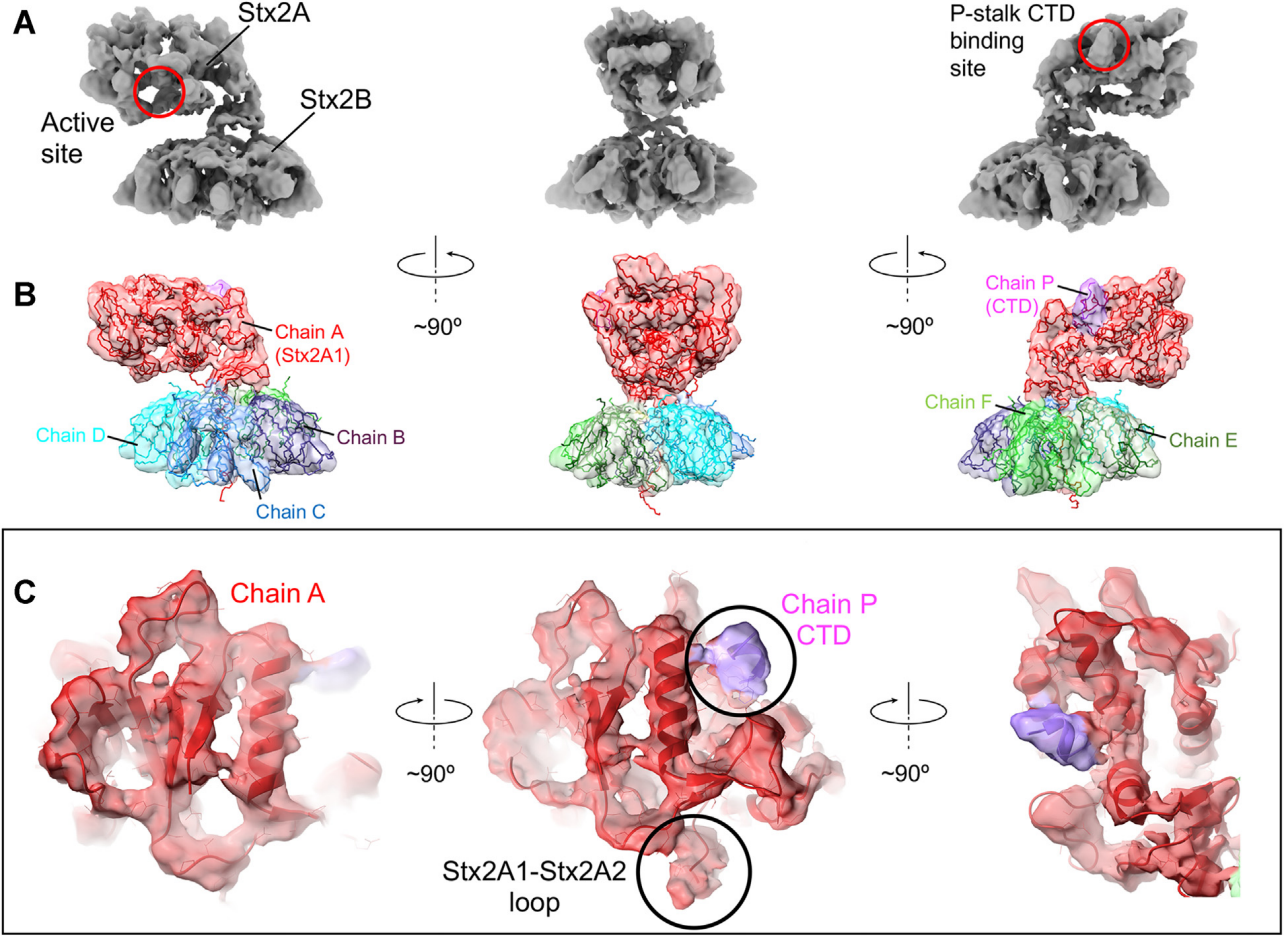
**Structural features of Stx2a holotoxin in complex with the ribosomal P-stalk.***A*, different views of the cryo-EM map of Stx2a–P-stalk reveal a monomeric Stx2A subunit bound to a pentameric Stx2B subunit. *Red circles* denote a catalytic *N*-glycosidase active site and the P-stalk’s C-terminal domain (CTD)–binding site, both located on the surface of Stx2A subunit. *B*, molecular model derived from the cryo-EM map. The map is colored according to each protein chain: *red* for chain A (*i.e.*, Stx2A composed of two fragments: Stx2A1 shown on the picture and Stx2A2, not shown, which intercalates into the pentameric ring formed by Stx2B) and chains B, C, D, E, and F forming a pentameric Stx2B (displayed in *purple*, *blue*, *cyan*, *dark green*, and *light green*, respectively). The chain P, shown in *violet*, is the six amino-acid fragment from the CTD of the P-stalk. *C*, different views of Stx2A (chain A, shown in *red*) in complex with the CTD (chain P shown in *violet* and indicated by a *black circle*). The loop connecting Stx2A1 and Stx2A2 fragments of Stx2A subunit is highlighted by a *black circle* in the *middle panel*. Stx2a, Shiga toxin 2a; Stx2A, A subunit of Stx2a.

Stx2–P-stalk samples were assessed by negative-stain EM. Resultant micrographs showed approximately 500 to 800 particles well dispersed across the field of view (Fig. S2), confirming that Stx2a–P-stalk is a good target for cryo-EM structure determination. To ensure the complex is formed on EM grids, we mixed both components at the concentration approximately 20 times higher than the *K*_*D*_ for the complex formation. For image processing and structure determination, we developed a novel processing pipeline by integrating four different software packages into a single platform (Fig. S3) (23). Details of the single particle analysis workflow are presented in Fig. S4. Representative micrographs and 2D class averages are shown in Figs. S4 and S5*A*, respectively. The angular distribution heatmap obtained for the initial model calculated in cryoSPARC v3 (Structural Biotechnology Inc.) confirms the complete coverage of the angular space (Fig. S5*B*). To ensure the resolution estimates are not affected by masking, the mask applied during refinements was not too tight, and it encompassed all parts of the map (Fig. S5*C*). The structure of the Stx2a–P-stalk complex shown in Figure 3*A* was refined to 4.1 Å resolution according to the 0.143 threshold from the Fourier shell correlation plot (Fig. S6*A*). The local resolution values range from 3.5 to 7.5 Å. A histogram showing a distribution of local resolutions along with the local resolution values mapped onto the surface of Stx2a–P-stalk are presented in Fig. S6, *B* and *C*, respectively. The molecular model of Stx2a–P-stalk, which was derived from the cryo-EM map using atomic coordinates of Stx2a (Protein Data Bank [PDB] ID: 6X6H) as a starting model, displays good validation statistics summarized in Fig. S7 and Table S1.

**Figure 4:**
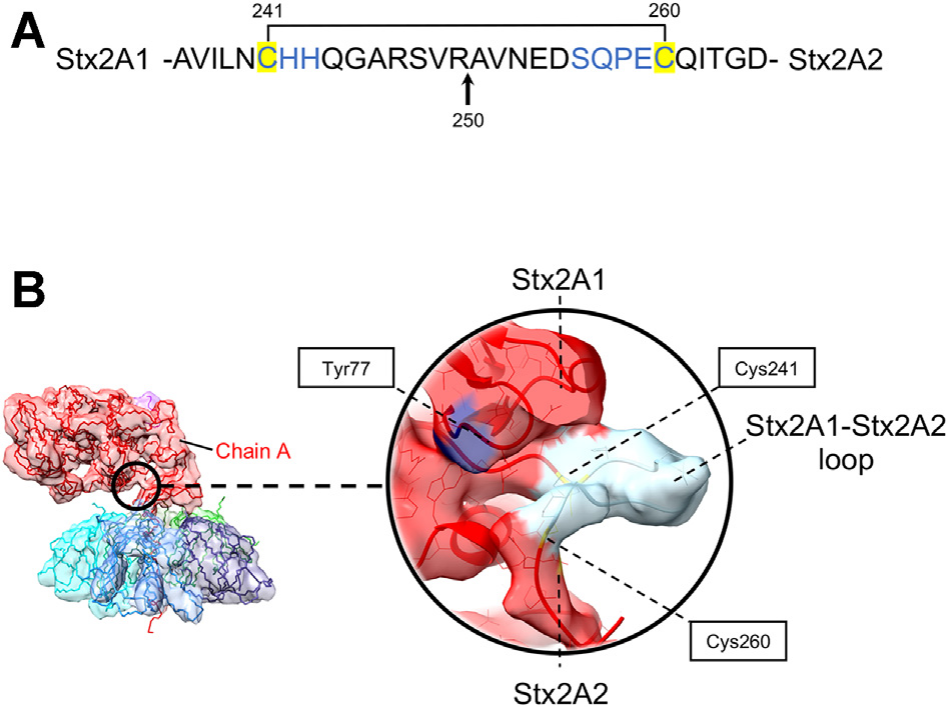
**Structural analysis of the loop connecting Stx2A1 and Stx2A2 fragments of Stx2A.***A*, the amino-acid sequence of the Stx2A1–Stx2A2 loop critical for the release of Stx2A1 from Stx2A2–Stx2B. The loop is stabilized by a disulfide bond, which is indicated in the figure. This disulfide bond involves residues Cys241 and Cys260 shown in *yellow boxes*. A cleavage site by furin protease at Arg250 is indicated by a *black arrow*. The loop forming amino acids, which have been modeled into the cryo-EM map, is highlighted in *blue*. These residues are Cys241–His243 and Ser256–Cys260. *B*, the density representing the Stx2A1–Stx2A2 loop in the cryo-EM structure of Stx2a–P-stalk is highlighted by a *black circle*. The cryo-EM map is colored according to the individual protein chains, as for Figure 3*B*. The *inset* shows structural details of the Stx2A1–Stx2A2 loop. The map density representing the loop is colored in *light blue*. Cys241 and Cys260, which form a disulfide bond, are also shown. An active site Tyr77, located in Stx2A1 and displayed in *purple*, is occluded by Stx2A2 prior to release of Stx2A1 from Stx2a. Stx2A, A subunit of Shiga toxin 2a.

The cryo-EM map presented in Figure 3*A* reveals densities representing a monomeric Stx2A subunit bound to a pentameric Stx2B subunit. Figure 3*B* displays atomic coordinates of Stx2a–P-stalk with the map colored according to the individual protein chains. Stx2A, denoted as chain A (Fig. 3*C*), is composed of two fragments, namely a catalytic Stx2A1 fragment consisting of the *N*-glycosidase active site and the P-stalk CTD-binding site (Fig. 3*A*), and Stx2A2 composed of an α-helix, which inserts into the channel formed by a pentameric ring of Stx2B (Fig. S8*D*). Stx2B is composed of five chains denoted as chains B, C, D, E, and F (Figs. 3*B* and S8, *B*–*D*). The structure reveals the presence of a density associated with Stx2a in the region identified earlier as the binding site for the CTD (Figs. 3*C* and S8*A*). This density can be fitted with the C-terminal six amino acids from a single P-protein of the P-stalk, denoted as chain P (Fig. 3, *B* and *C*). Interestingly, the density corresponding to a flexible loop connecting StxA1 and StxA2 is visible in the cryo-EM structure (Figs. 3*C* and 4*B*). The cryo-EM structure of Stx2a holotoxin shows an overall good agreement with previously determined X-ray structures of Stx2a (PDB ID: 1R4P, (26)) and Stx2a–P11 (PDB ID: 6X6H (20)) (Fig. S9). However, unlike crystal structures, the cryo-EM data reveal an extensive dynamic behavior of Stx2a–P-stalk, including conformational changes in the P-stalk–binding site upon complex formation (Movie S1).

### The cryo-EM structure of the Stx2a–ribosomal P-stalk complex shows a part of the loop connecting Stx2A1 and Stx2A2

The loop connecting Stx2A1 and Stx2A2 consists of the furin cleavage site at Arg250 (Fig. 4*A*). This loop has not been visualized in any of the crystal structures of Stx2a and Stx2a–P11 (20). The cryo-EM structure reveals a part of this loop spanning residues Cys241–His243 and Ser256–Cys260 (Fig. 4). The structure shows that Tyr77, located in the *N*-glycosidase active site, is occluded by Stx2A2 (Fig. 4*B*). Thus, the proteolytic cleavage at Arg250 is responsible for activation of Stx2A1 by removing the steric hindrance imposed by Stx2A2 binding. In addition, the structure shows that the loop is stabilized by the disulfide bond involving residues Cys241 and Cys260 (Fig. 4). This disulfide bond is reduced in the ER, triggering the release of Stx2A1 from Stx2a.

### The binding interface between Stx2a and the CTD of the ribosomal P-stalk

**Figure 5:**
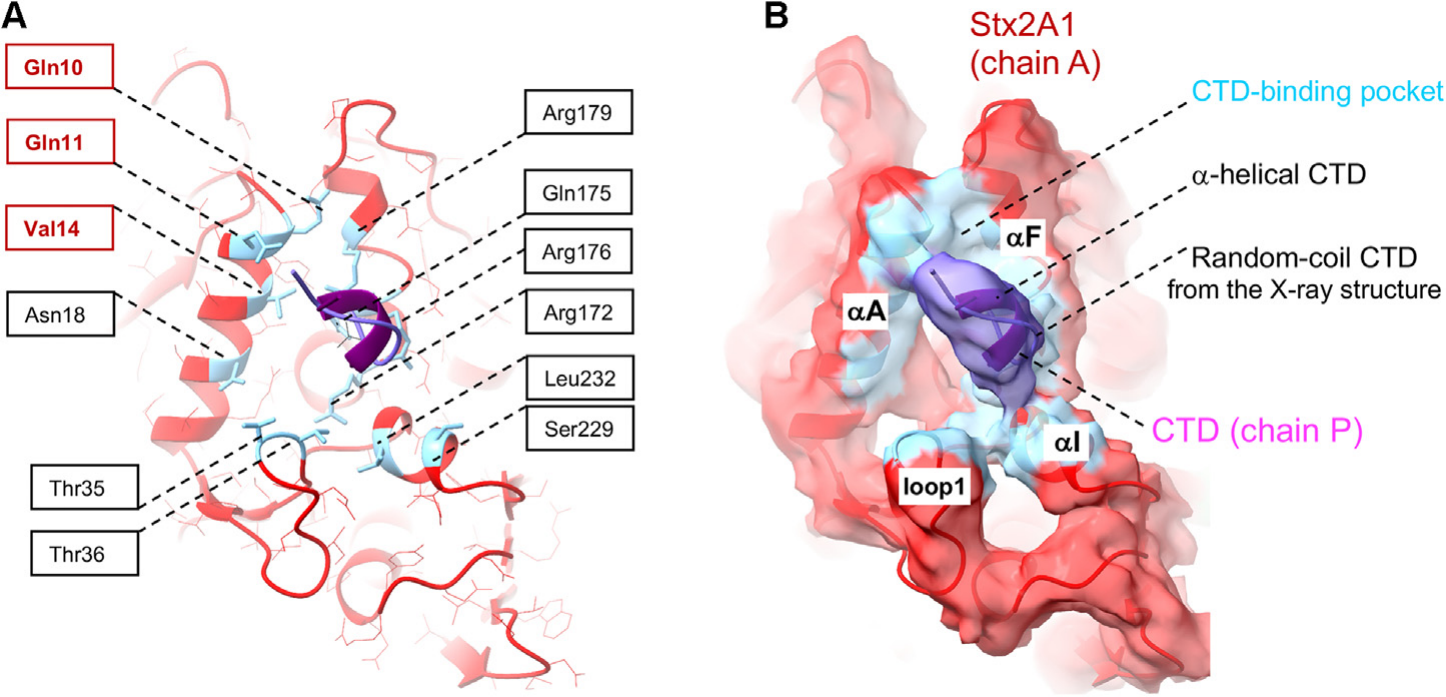
**The CTD-binding pocket on the surface of Stx2A1.***A*, Sta2A1 amino acids involved in binding to the six amino-acid peptide from the CTD of a single P-protein (shown in *violet*) are displayed as *blue sticks*. Some of these amino acids have already been implicated in the interaction with Stx2A1 by the crystal structure of Stx2a–P11 (20). These residues are highlighted by the *black boxes*. In contrast, the cryo-EM structure reveals a number of novel residues involved in binding to the P-stalk. These, so far, identified amino acids Gln10, Gln11, and Val14, are indicated by *red boxes*. *B*, the surface representation of the P-stalk–binding pocket of Stx2A1 (chain A). The density representing the P-stalk’s CTD (chain P, shown in *violet*) can be fitted with the hexapeptide from the CTD adopting either the random-coil or α-helical conformations. Both random coil and α-helical hexapeptides are displayed and labeled in the figure. The CTD-binding pocket is formed by α-helices A, F, I, and loop 1, all displayed in *light blue* and labeled in the figure. CTD, C-terminal domain; Stx2A, A subunit of Stx2a.

The binding interface between Stx2a and the CTD of the ribosomal P-stalk is displayed in Figure 5*A*. The CTD-binding pocket is located on the opposite side of the Stx2a surface than the active site, and it is formed by α-helices: A, F, I, and loop 1 spanning residues Pro27–Ser39 (Fig. 5*B*). The buried surface area includes 797 A^2^ of the CTD (chain P) and 17,010 A^2^ of Stx2A1 (chain A) (Fig. 5*B*). The binding interface involves Stx2a residues previously implicated in the P11 binding by the X-ray structure (20) and mutational analysis (27), such as Asn18, Thr35, Thr36, Ser229, Leu232, and the conserved Arg172, Arg176, and Arg179 (Fig. 5*A*). Importantly, the cryo-EM structure reveals novel previously unidentified amino acids, involved in binding to the CTD, namely Gln10, Gln11, and Val14 (Fig. 5*A*).

The cryo-EM structure unveils that the six amino-acid peptide (GFGLFD) from the CTD interacts with Stx2a. Because local resolutions are in the range of 4.5 to 6.5 Å (Fig. S6*C*), a full atomic model of the hexapeptide cannot be derived from the cryo-EM map directly. However, the density representing the peptide overlaps with atomic coordinates of all six amino acids from P11 visualized by the crystal structure of Stx2a–P11 (20) (Fig. 5*B*), with the backbone deviation ranging from 1.4 to 2.5 Å. The hydrogen bonding pattern involving Gln175 and Asp11, and Arg176 and Phe10 from Stx2a and the CTD, respectively, is satisfied in both the X-ray and the cryo-EM structures. In contrast to the X-ray structure, Val14 forms nonpolar interactions with the first N-terminal Phe7 from the CTD (Fig. 5*A*).

The density representing the CTD in the cryo-EM structure can also be fitted with the six amino-acid peptide adopting an α-helical, rather than a random-coil conformation, as observed in the X-ray structure (Fig. 5*B*). Interestingly, fitting the density with a short α-helix satisfies the hydrogen bonding pattern described previously. The observation that the CTD might behave as a random coil or as an α-helix is of significance, and it indicates the possibility for structural rearrangements in this region, as indicated previously (28).

To access whether the binding of Stx2a to the entire ribosome, including the P-stalk, would stabilize the CTD–Stx2a interaction such that a fragment of the P-stalk reaching beyond the C-terminal six amino acids can be imaged, we attempted to determine a structure of Stx2a complexed with the 3 MDa *S. cerevisiae* ribosome and to visualize details of the CTD–Stx2a interface by focused refinements. We solved a cryo-EM structure of the 80S ribosome (Fig. S10), but we were not able to visualize Stx2a binding because of the intrinsic flexibility of the P-stalk (Fig. S10*B*).

### Modeling and structural analysis of the ribosomal P-stalk pentamer

**Figure 6:**
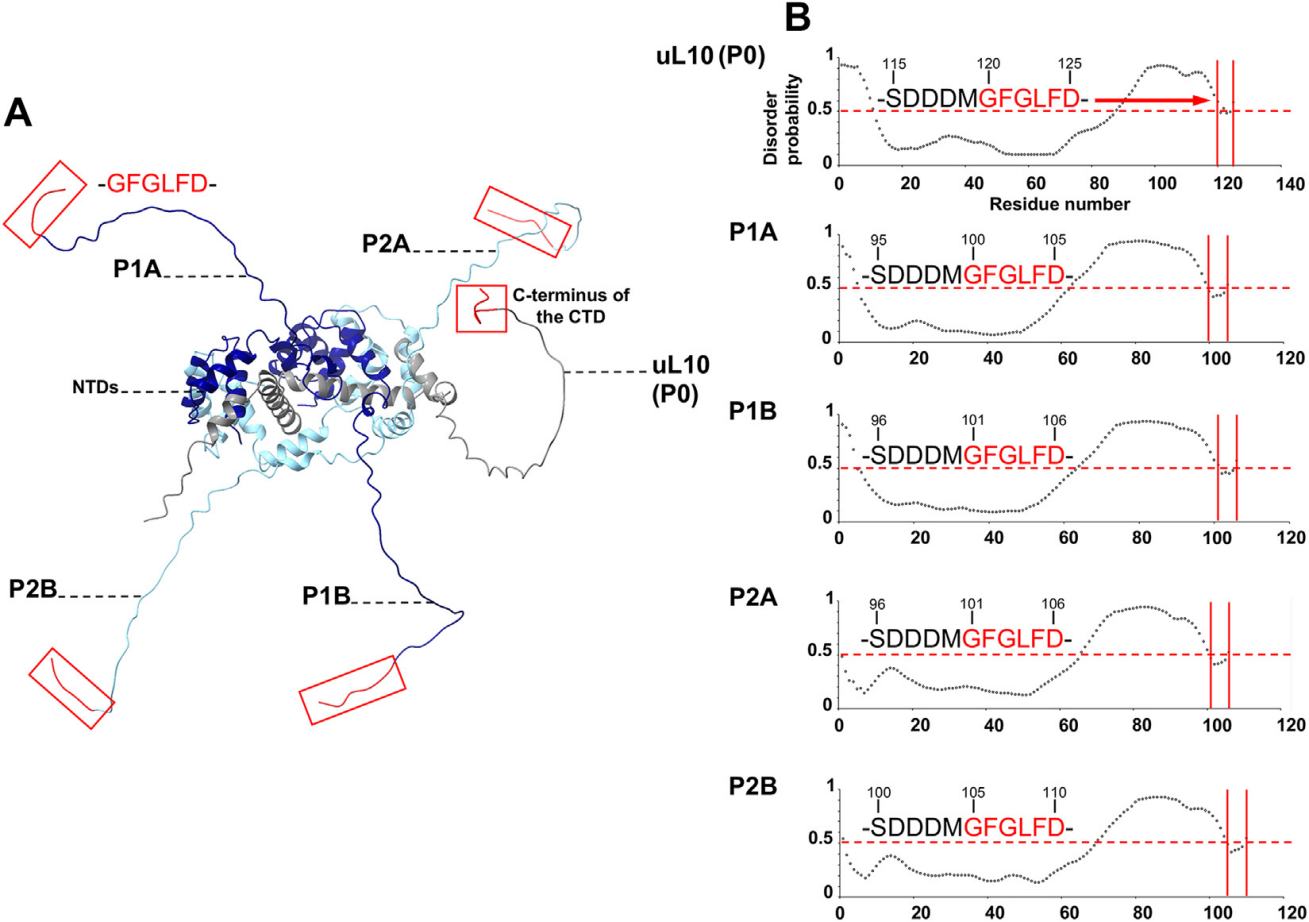
**Structure prediction of the ribosomal P-stalk pentamer.***A*, a structure of the pentameric P-stalk consisting of ul10 (P0), P1A, P1B, P2A, and P2B subunits was predicted with AlphaFold2 (53). The structure is characterized by high-predicted local distance difference test (lDDT) values (Fig. S11*B*), and it agrees with the NMR structure of the P1A–P2B dimer (Protein Data Bank ID: 4BEH) displayed for Fig. S12 (30). The uL10 (P0) is displayed in *gray*, P1A and P1B are shown in *dark blue*, and P2A and P2B are displayed in *cyan*. Unstructured C-terminal domains (CTDs) of the P-proteins are linked to the 20-helix bundle formed by the N-terminal domains (NTDs). The six amino-acid peptides from the C-terminal ends of the P-proteins (GFGLFD), which were implicated in biding to Stx2a by the cryo-EM structure, are highlighted by *red boxes*. *B*, the disorder probability analysis performed for the P-proteins in the program PrDOS (University of Tokyo) (54). Plots show the disorder probability coefficients plotted as a function of a residue number for a given P-protein. Disorder probability coefficients below the value of 0.5 (shown as *red horizontal lines*) indicate structured regions in the P-proteins. The analysis is consistent with the structure prediction of the P-stalk (Fig. 6*A*), as the regions corresponding to the NTDs are characterized by low values of the coefficients. The plots show that all but the last six amino acids from the C termini of the P-proteins are unstructured, as indicated by the *red arrow*. The 11 amino-acid sequences, including these hexapeptides, are shown in the figure. Disorder probability coefficients representing these hexapeptide sequences highlighted in *red* and indicated by *red lines* on the plots suggest folded regions. The coefficient values are close to the transition point between order and disorder, highlighting the propensity of the hexapeptide sequences for secondary structure formation. This result is consistent with the results of docking of random-coil and α-helical hexapeptides from the CTD to the cryo-EM map of Stx2a–P-stalk, shown for Figure 5*B*. Stx2a, Shiga toxin 2a.

The ribosomal P-stalk is a dynamic structural feature of the ribosome, which facilitates interactions of the ribosome with trGTPases and RIPs (29). A part of the P-stalk, namely the CTD, can be regarded as an intrinsically disordered protein (IDP). In support of this notion, the NMR analysis demonstrated that the CTDs of P-proteins are disordered in solution (30, 31). Consequently, the conformational rearrangements occurring upon binding of an RIP to the P-stalk pose a considerable challenge for structural analysis. We have thus employed AlphaFold2 (Deep Mind) for structure prediction of the P-stalk including a truncated uL10 analogous to the one used for cryo-EM studies, which contained an intact P-domain, along with P1A, P1B, P2A, and P2B subunits (Figs. 6*A* and S11).

The predicted structure of a fully assembled P-stalk reveals a cuboid shape with a diameter of approximately 70 Å × 40 Å × 40 Å corresponding to the folded NTDs of the P-stalk on one end and an extended unstructured region representing the CTDs on the other end (Figs. 6*A* and S11*A*). The structural core of the P-stalk is formed by the four α-helices from uL10 (P0), which provide an extended scaffold for binding by the 4-helix bundles from NTDs of each individual P-protein subunit (P1A, P1B, P2A, and P2B). A modeled structure of the P-stalk is in good agreement with the NMR structure of the individual P1–P2 dimer (PDB ID: 4BEH) (30) and also with the P-stalk fragment revealed by the structure of the ribosome (PDB ID: 4V6X) (14). The backbone RMSDs calculated for the NMR structure and the AlphaFold2 model are 1.2 and 0.9 Å in the NTDs of P1 and P2 subunits, respectively (Fig. S12). The CTDs of P1 and P2 are extended approximately ∼125 Å away from the NTDs in the NMR structure, and they are unfolded in solution (Fig. S12).The model reveals that the C-terminal six amino-acid peptides from the CTDs (sequence GFGLFD), which are implicated in binding to Stx2a by the cryo-EM structure, remain unstructured in the absence of the toxin (Figs. 6*A* and S11*A*). To assess the propensity for the secondary structure formation in these regions, we carried out the disorder probability analysis presented in Figure 6*B*. The analysis revealed that, in contrast to the reminder of the CTD, the six amino-acid sequences from the C terminus of each P-protein have a predisposition for secondary structure formation. This result is consistent with the results of the CTD docking into the cryo-EM map (Fig. 5*B*).

### Analysis of the intrinsic dynamics of the Stx2a–P-stalk interaction

**Figure 7:**
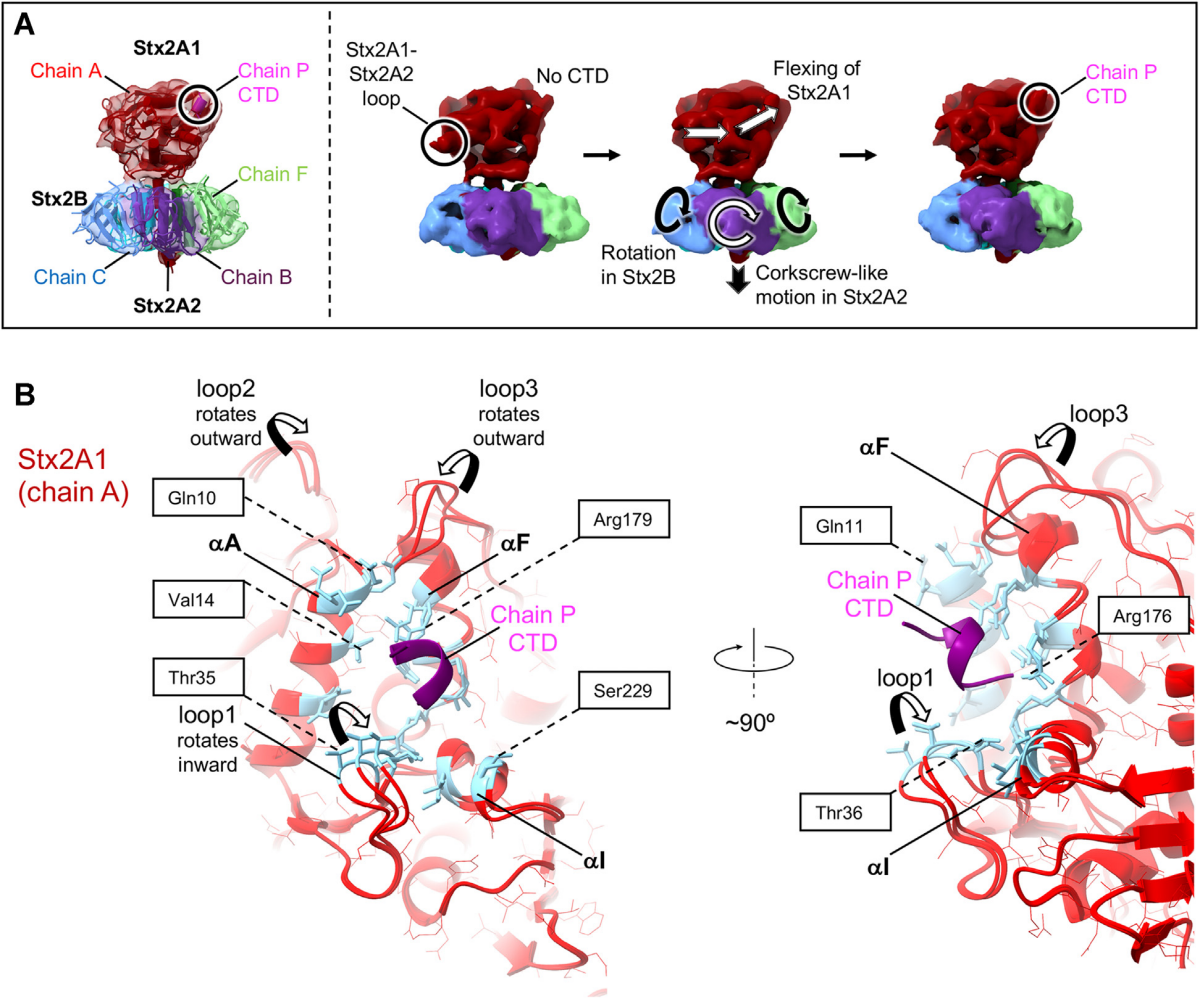
**Dynamic behavior of the Stx2a–P-stalk complex and structural rearrangements occurring upon complex formation.***A*, the molecular motion in Stx2a–P-stalk revealed by the PCA of the cryo-EM data. The cryo-EM map is color coded according to the individual protein chains. Stx2A1 and Stx2A2 fragments of Stx2A forming chain A are shown in *red*. Stx2B chains B, C, and F are colored in *purple*, *blue*, and *light green*, respectively. The CTD peptide is displayed in *violet*. The CTD-binding site on Stx2A1 and the Stx2A1–Stx2A2 loop are highlighted in *black circles*. The PCA reveals that Stx2A1 displays molecular flexing indicated by *white arrows*. The motion in Stx2A1 is accompanied by the rotation-like movement of the individual Stx2B subunits (indicated by *circular arrows*) and a corkscrew-like motion of Stx2A2 (indicated by the *black arrow*). The appearance of the density representing the CTD correlates with the disappearance of the density corresponding to the Stx2A1–Stx2A2 loop. Results of the PCA are described in details in the text (Fig. S13) and visualized in Movie S1. *B*, a superposition of the CTD-binding pockets on the surface of Stx2A1 from the three structural snapshots of Stx2a–P-stalk shown in *A*. Stx2A1 and the CTD are displayed as *red* and *purple ribbons*, respectively. The CTD binding is accompanied by structural rearrangements in α-helices A, F, I, and in loops 1, 2, and 3 indicated in the figure by *arrows*. The figure displays some of the residues implicated in the CTD binding. These residues are shown as *blue sticks*. Please compare with Figure 5*A* and see text for more details. CTD, C-terminal domain; PCA, principal component analysis; Stx2A, A subunit of Shiga toxin 2a.

The cryo-EM structure of Stx2a–P-stalk and structural modeling of the ribosomal P-stalk pentamer indicated the propensity for conformational rearrangements in the CTD upon complex formation. We have therefore performed the principal component analysis (PCA) of the cryo-EM data to directly assess the mode of the Stx2a–P-stalk interaction (Figs. 7 and S13). The initial set of 1,401,983 particle images, obtained after 2D classification and averaging, was used for the PCA (Fig. S4). This stack of particles was not subjected to the 3D classification or 3D refinements. The PCA reveals an intrinsic dynamics of the complex, the fact supported by an extensive variability in the three calculated eigenvectors (Fig. S13*A*), and by a nonuniform appearance of the reaction coordinate plots (Fig. S13*B*). The continuous distributions of reaction coordinates on these plots are indicative of the continuous motion in Stx2a–P-stalk, rather than the presence of discrete conformations. The intrinsic dynamics of the Stx2a–P-stalk complex, characterized by the first eigenvector, is summarized in Figure 7*A* and visualized in Movie S1.

Stx2A1 displays molecular flexing, the motion accompanied by the rotation-like movement of the individual Stx2B subunits and a corkscrew-like motion of Stx2A2 (Fig. 7*A*). The flexing of Stx2A1 affects the conformation of the P-stalk–binding pocket. Structural changes occurring upon the P-stalk binding are summarized in Figure 7*B*. The inward rotation of loop 1 (Pro27–Ser39) toward the CTD brings residues Thr35 and Thr36 into the binding pocket and indirectly influences the position of the α-helix I harboring active-site residues Ser229 and Leu232. The mobility of loop 2 spanning amino acids Ile54–Leu67, which rotates outward from the CTD-binding site, indirectly affects the position of the α-helix A containing the CTD-binding residues Gln10, Gln11, and Val14. The movement and outward rotation in loop 3 (residues Leu182–Pro187) affect the orientation of the α-helix F consisting of the three conserved arginine residues (Arg172, Arg176, and Arg179), notably by bringing Arg179 into the close proximity to the CTD. Conformational rearrangements described previously bring residues from Stx2a binding pocket into contact with the CTD to stabilize the Stx2a–P-stalk interaction (Fig. 7 and Movie S1).

## Discussion

We applied cryo-EM in combination with SPR and computational methods to investigate the structure and dynamics of the interaction between Stx2a and its native binding partner, the ribosomal P-stalk. Cryo-EM provides the most complete structure of Stx2a to date (Fig. 3*A*) and shows that Stx2a is anchored to the P-stalk *via* a short peptide from the CTD of a single P-protein (Fig. 5). Importantly, for the first time, the cryo-EM analysis reveals a remarkable dynamic behavior of the Stx2a–P-stalk complex including molecular flexing of Stx2A1, accompanied by the rotation-like movement of the individual Stx2B subunits, and a corkscrew-like motion of Stx2A2 (Fig. 7*A* and Movie S1). These structural changes are likely involved in the release of Stx2A1 from Stx2a.

Biochemical studies of RIP interactions with short peptides mimicking the CTD reported low binding affinities with the *K*_*D*_ values in the micromolar range, demonstrating that short peptides do not represent optimal binding partners for RIPs. Consistent with these results, the *K*_*D*_ measured for the interaction of Stx2a with P11 is 48 μM (Fig. S1*C*). In contrast, the ribosomal P-stalk binds to Stx2a with the *K*_*D*_ of 823 nM (Fig. 2*F*). Approximately 60-fold difference in binding affinities suggests that the native P-stalk allosterically contributes to stabilization of the interaction with Stx2a (Fig. 6). In support of this notion, by using computational and cryo-EM analyses, we observed conformational changes in the P-stalk–binding site of Stx2a triggered by binding to the CTD (Fig. 7*B*). Such a mode of interaction resembles the induced fit mechanism characteristic for IDPs. These structural rearrangements include changes in α-helices A, F, I, and loops 1, 2, and 3 (Fig. 7*B*). They collectively bring Stx2a residues involved in binding into contact with the CTD to lock the CTD on Stx2A1 (Fig. 7*B*).

The cryo-EM structure, for the first time, shows a part of the loop connecting Stx2A1 and Stx2A2 (Fig. 4), which is directly involved in processing of the holotoxin within the host cell lumen. A proteolytic cleavage of the Stx2A1–Stx2A2 loop is vital for the activation and the cytotoxicity of Stx2a. The majority of the residues from this loop (His243–Pro258) are missing in the previous crystal structures of Stx2a (20, 26). The cryo-EM structure shows that the loop is solvent exposed, explaining its susceptibility to protease degradation. In support of this observation, the results of PCA (Fig. 7*A* and Movie S1) and 3D classification (Fig. S4) revealed a fraction of molecules without the well-defined density representing the loop. Interestingly, the molecular flexing detected in Stx2A1 correlates with the disappearance of the density representing the Stx2A1–Stx2A2 loop (Fig. 7*A* and Movie S1).

**Figure 8:**
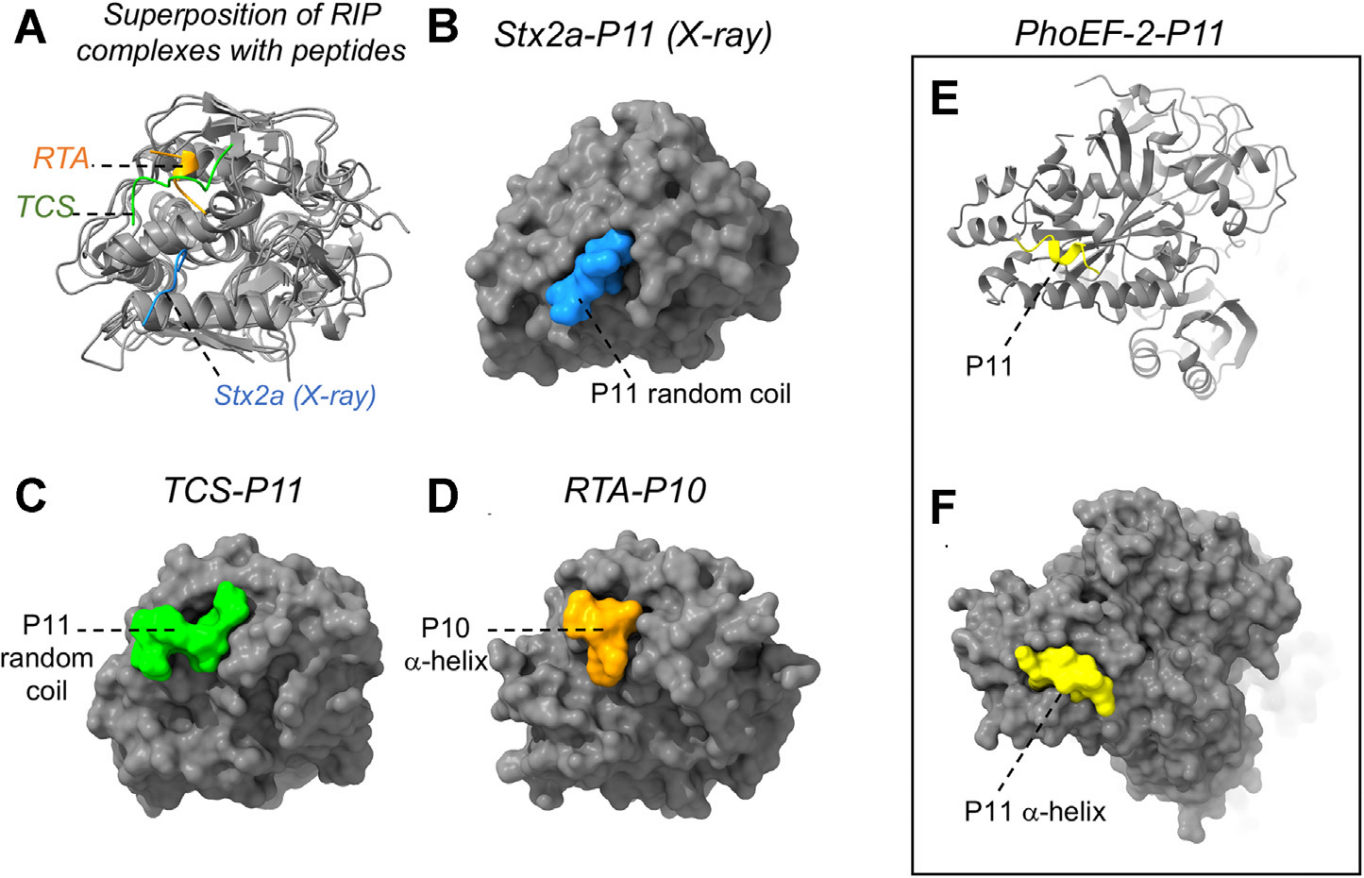
**The binding interfaces formed by the C-terminal peptides from the CTDs of P-proteins with RIPs and trGTPases.***A*, structural alignment of RIPs with the CTD peptides. All proteins are displayed in *gray*. The peptides are displayed in different colors and described in the figure. The following *ribbon* diagrams are displayed: (i) Stx2a–P11 (Protein Data Bank [PDB] ID: 6X6H) determined by X-ray crystallography. The six random-coil amino acids from the C terminus of P11 are shown in *blue*, (ii) TCS bound to P11 (PDB ID: 2JDL). The P11 complexed with TCS is displayed in *light green*, and (iii) RTA in complex with P10 (PDB ID: 5DDZ). The P10 is highlighted in *orange*. Surface representations of (*B*) Stx2a–P11, (*C*) TCS–P11, and (*D*) RTA–P10. Please see text for the description of the intermolecular interfaces displayed in the figure. *E*, a ribbon diagram of the crystal structure of the elongation factor PhoEF-2 bound to P11 (displayed in *yellow*), (PDB ID: 5H7L). *F*, the surface representation of PhoEF-2–P11 shows that the peptide adopts the α-helical conformation in complex with trGTPase. CTD, C-terminal domain; RIP, ribosome-inactivating protein; RTA, ricin toxin A; TCS, trichosanthin; trGTPase, translational GTPase.

The changes in the structure of Stx2a are likely accompanied by the conformational rearrangement within the C-terminal six amino acids of the CTD upon binding. The crystal structures of RIP complexes with the peptides mimicking the C-terminal 10 (P10) or 11 (P11) amino acids from the CTD revealed that these peptides can adopt different conformations. The structural alignment of different RIPs complexed with the CTD peptides is presented in Figure 8*A*. In the crystal structures of Stx2a–P11 (in which only six amino acids from the C terminus of P11 are visible) (20) and trichosanthin (TCS)–P11 (32), the CTD peptides adopt a random-coil configuration (Fig. 8, *B* and *C*, respectively). In contrast, the crystal structure of ricin toxin A (RTA) bound to P10 (33) shows that the C-terminal six residues of P10 resembles an α-helical turn (Fig. 8*D*). The density representing the CTD in the cryo-EM map of Stx2a–P-stalk can be fitted either with the random coil or α-helical hexapeptides (Fig. 5), possibly suggesting a mixture of both conformations in the sample. This finding implicates a random coil to an α-helix transition in the CTD upon binding to Stx2a. This observation is supported by the molecular dynamics simulation of the TCS–CTD interaction, which indicated that the CTD has the propensity for α-helix formation in the presence of TCS (28).

The presence of the whole P-stalk structure may be required for such a conformational transition to occur. Consistent with this notion, the AlphaFold2 model of the full-length P-stalk shows extended and unstructured CTDs linked to the folded NTD core in the absence of the toxin (Figs. 6*A* and S11*A*). In addition, the computational analysis indicates that, unlike the reminder of the CTD, the last six amino acids from the CTD of the P-proteins have the propensity for the secondary structure formation, suggesting a random coil to α-helix transition upon Stx2a binding (Fig. 6*B*). In agreement with this notion, we showed in previous studies that RTA binds to the P-stalk pentamer with higher affinity than it does to the trimeric P-stalk comprising of uL10 and one copy of the P1–P2 heterodimer (34). This result demonstrates that a fully assembled P-stalk complex stabilizes the interaction with an RIP. The model for the allosteric mode of the interaction between Stx2a and the P-stalk, which we propose, corroborates the previously proposed model for the trGTPase–P-stalk binding. In the latter model, a short six or seven amino-acid peptide corresponding to the C terminus of archaeal P-proteins folds into the α-helix upon binding to aEF1A, aIF5B, or aEF2 (35, 36). The crystal structure of PhoEF-2 bound to P11 reveals that a part of P11 forms an α-helix in complex with the elongation factor (Fig. 8, *E* and *F*).

The P-stalk–binding pockets of Stx2a, RTA, and TCS are structurally diverse. Stx2a has a shallow pocket lined by mainly positively charged residues at one site and the hydrophobic residues at the other side (Fig. 5*A*) (20), whereas TCS (32) and RTA (33) have deep hydrophobic pockets at different locations than Stx2a (Fig. 8, *C* and *D*, respectively). The observed differences in respect to protein–protein interfaces formed upon binding of RIPs to the CTD may indicate that, because of the IDP-like properties of the ribosomal P-stalk, the CTD is used in a versatile way by different binding partners, with the C-terminal six residues playing a critical role for the interaction (36, 37, 38).

Recently, high-speed atomic force microscopy was used to visualize the interaction of the archaeal heptameric P-stalk attached to the *E. coli* ribosome with eEF1 and eEF2 (39). The high-speed atomic force microscopy study indicated the role of the P-stalk in recruitment of trGTPases onto the ribosome. Multiple copies of eEF1 and eEF2 were bound to the P-stalk at any given time increasing the local concentration of trGTPases available for loading onto the ribosome. In an analogous manner, the P-stalk has the capacity to bind up to five copies of Stx2a. Because all Stx2a–CTD interfaces are equivalent, cryo-EM images representing individual binding sites are averaged during 2D and 3D classification and averaging, yielding a final structure. In support of this notion, the SPR analysis of the Stx2a–P-stalk binding reveals that the interaction fits into a 1:1 kinetic model, indicating that there is only one type of the interaction between Stx2a and the P-stalk. However, the stoichiometry of binding between Stx2a and the P-stalk may vary, with multiple Stx2a molecules associating with the P-stalk pentamer at any given time, each molecule interacting with one of the five CTDs. Because these interactions occur simultaneously with similar kinetics, they are indistinguishable by SPR.

While structure determination by cryo-EM is now becoming a method of choice for an increasing number of investigators, successful application of cryo-EM to structure determination of 72 kDa Stx2a complexed with a six amino-acid peptide represents a formidable task. The structure described here is one of the few structures representing biomolecules with molecular weights below 100 kDa deposited to date to the Electron Microscopy Data Bank and the PDB (21).

Because of the high potency of RIPs and the lack of effective therapies neutralizing their cytotoxic effects, the active sites of RIP have already been targeted for discovery of inhibitors by a structure-based drug design. However, targeting the ribosome-binding site of RIPs provides an attractive alternative with the potential for the development of the combination therapy. Recently, we have identified small-molecule inhibitors binding to the ribosome-binding site of RTA and established the CTD-binding pocket of RTA as a new drug target (40, 41). Although the identified compounds bind remotely from the active site of RTA, they inhibit the SRL depurination activity mediated by the ricin holotoxin (40, 41). Likewise, the ribosome-binding site of Stx2a is a convenient, yet unexplored, drug target. The cryo-EM structure provides an important insight into the dynamics of the Stx2a–P-stalk interaction and identifies residues at the binding interface as potential targets for drug discovery. Understanding the conformational rearrangements in Stx2a–P-stalk is critical for the design of selective small-molecule inhibitors directed toward the CTD-binding site of Stx2a for the treatment of STEC infections.

## Experimental procedures

### Isolation of the ribosomal P-stalk

The purification of the P-stalk complex was performed using *S. cerevisiae* uL10 mutant uL10TH199 (MATa; RPP0-TH199; his3Δ1; leu2Δ0; lys2Δ0; and ura3Δ0), as described previously (42). In brief, a short amino acid sequence recognized by thrombin was introduced into uL10 at position 199. A truncated uL10 (amino acids 199–312) contained an intact P-domain, which binds to the P1A–P2B and P1B–P2A dimers (ΔuL10199-312-(P1A–P2B)-(P1B–P2A)) (42). The P-stalk complex was purified to homogeneity using affinity and size-exclusion chromatography (25). Purity of the ribosomal stalk complexes was verified by 14% SDS–PAGE, 10% native PAGE, and native mass spectrometry. Stx2a holotoxin was purchased from Phoenix Laboratory.

### Biacore analysis of Stx2a interaction with the ribosomal P-stalk and P11

The Biacore 8K+ (Cytiva) was used to study the interaction. Stx2a was immobilized on Fc2 of a CM5 chip in four different channels using amine coupling. Fc1 was activated and blocked. The P-stalk was flown over the surface of the chip at different concentrations listed in Figures 2*E* and S1*B*, at a flow rate of 30 μl/min, for 30 s and then left to dissociate for additional 60 s. The running buffer contained 10 mM Hepes (pH 7.4), 150 mM NaCl, 10 mM MgCl_2_, 3 mM EDTA, and 0.05% P20. Because of the fast on/off kinetics, the dissociation constants (*K*_*D*_) for the interactions were determined by fitting the binding data at steady state using Biacore Insight Evaluation Software 3.0 (Figs. 2*F* and S1*C*).

### Negative-stain EM

Samples of individual proteins and protein complexes were screened by negative-stain EM at the total protein concentration in the range of 0.005 to 2.1 mg/ml in 50 mM Hepes buffer (pH 7.5) containing 150 mM NaCl and 10 mM MgCl_2_. Samples were incubated for 10 min at room temperature prior to EM analysis. The 3 μl aliquots were applied to carbon-coated 300-mesh copper grids, which were previously rendered hydrophilic with PELCO easiGlow glow discharger. Grids were stained with 2% (w/v) uranyl formate for 3 min at room temperature. After staining, grids were air dried and inserted into the microscope. Images were recorded using Philips CM-12 electron microscope operating at 80 kV acceleration voltage at a magnification of 60,000 or 75,000 times corresponding to the pixel size of 1.95 Å/px or 2.44 Å/px, respectively.

### Cryo-EM sample preparation, screening, and data collection

Samples of Stx2a–ribosomal P-stalk were prepared by mixing individual proteins in buffers and concentrations described in the previous section. Samples used for final data collection consisted of 2.1 mg/ml complex obtained after mixing Stx2a:ribosomal P-stalk in 1:3 stoichiometry. Samples were adsorbed onto freshly glow-discharged 300 mesh gold Quantifoil R2/1 grids with PELCO easiGlow glow discharger and flash frozen in liquid ethane using either Vitrobot Mark IV dual-blotting plunger or Leica GP Climate-controlled sample plunger with a controlled temperature and humidity. The grids were screened with a 200 kV Thermo Fisher Scientific Talos Arctica electron microscope equipped with a Gatan BioQuantum energy filter and Gatan K2 Summit direct electron detector at the Center for Integrative Proteomics Research at Rutgers University using the SerialEM (University of Colorado, Boulder) software (27) or EPU (ThermoFisher Scientific) software for automated data collection. We used the following acquisition parameters: dose rate of 5.3 e^−^/px/s in a counting mode, magnification of 130,000 times corresponding to the pixel size of 1.037 Å/px with a defocus range −2.4 to −3.7 μm. We collected 40 frames with 200 ms per frame exposure, a total exposure of 8 s, and an accumulated dose of 42.4 e^−^. Prescreened grids containing the ribosomal P-stalk–Stx2a complex were shipped to the National Center for CryoEM Access and Training at New York Structural Biology Center for data collection with 300 kV Thermo Fisher Scientific Titan Krios, equipped with an energy filter and Gatan K3 direct electron detector. The data were acquired using Leginon software (43) for automated data collection. At the New York Structural Biology Center, the following acquisition parameters were used: dose rate of 29.41 e^−^/px/s in a super-resolution mode, magnification of 105,000 times corresponding to the pixel size of 0.4124 Å/px with a defocus range −0.8 to −2.5 μm. We collected 40 frames with 299 ms per frame exposure, a total exposure of 2 s, and an accumulated dose of 58.82 e^−^.

### Structure determination, model building, and refinements

A structure of the complex between ribosomal P-stalk and Stx2a was calculated using cryoSPARC v3 (Structural Biotechnology Inc.) (44), RELION-3 (MRC Laboratory of Molecular Biology) (45), Scipion-3 (Spanish National Centre for Biotechnology) (46, 47), and Phenix 1.18 (University of Cambridge,  Duke University, LANL, LBNL) (48). Thirty-five thousand and fifty-two movies were patch motion– and patch contrast transfer function (CTF)–corrected in cryoSPARC v3. During motion correction, movies were binned by a factor of 2. Following inspection of preprocessed averaged images, 7989 images were discarded and 27,063 were used for particle picking. We manually picked 500 particles to create 2D templates for automated picking. The template picker automatically selected 5,024,944 particle images. Picked particles were inspected and extracted from averaged images with a box size of 300 pixels, 0.8248 Å/px, and subjected to three rounds of 2D classification and averaging. During 2D analysis, artifacts and particles not converging to stable classes were removed from the dataset. The resultant set of 1,401,983 particle images was used for *ab initio* modeling. The initial model was used as input for multiple rounds of homogenous and nonuniform refinements, during which additional 685,987 particle images were discarded. All calculated maps had C1 symmetry. Calculations in cryoSPARC v3 resulted in 6.8 Å map calculated with 715,996 particles. In the following steps, we used PyEM and in-house scripts to convert and transfer particle coordinates, used to calculate the 6.8 Å map, from cryoSPARC v3 to RELION-3. Prior to the transfer of coordinates, movies were imported to RELION-3, frames aligned with MotionCorr2 (UCSF) software to correct for beam-induced specimen motion, and CTF parameters were estimated with CTFFind4 (UMass Medical School). Particles were re-extracted from averaged images with a box of 300 pixels and subjected to additional five rounds of 2D classification and averaging, during which 514,346 particles were discarded. The resultant set of 201,650 particles was used for 3D classification with three classes. To avoid any potential model bias, an unsharpened *ab initio* map, which was initially used to calculate a 6.8 Å map in cryoSPARC v3, was imported to RELION-3, low pass-filtered to 30 Å, and used as a starting model. One of the three 3D classes, consisting of 112,924 particle images, revealed the additional density in the CTD-binding pocket on the surface of Stx2a; this structure was used for further refinements. We carried out the following refinements: CTF refinements (beam tilt, anisotropic magnification, per-particle defocus, an dper-micrograph astigmatism), Bayesian polishing of particles, multibody refinement, and postprocessing with *B*-factor sharpening and modulation transfer function correction. In addition, we carried out multibody refinements. UCSF Chimera (49, 50) was used to divide the input map into segments corresponding to Stx2A and Stx2B subunits and to create masks. Resolution of the final map was calculated using the gold standard Fourier shell correlation method according to the 0.143 criterion. In addition, we employed a deep-learning algorithm, DeepRes (51), available through Scipion-3 (46, 47) to calculate local resolutions. The molecular model was derived from the EM map with a combination of Phenix 1.18 (48) and COOT 0.9 (MRC Laboratory of Molecular Biology) (52) using atomic coordinates of Stx2a (PDB ID: 6X6H) as a starting model. The model was refined in Phenix 1.18, with imposed noncrystallographic symmetry, secondary structure, and Ramachandran restrains. The model was validated using MolProbity (Duke University). The refined model displays good validation statistics presented in Table S1.

### The PCA and structure predictions

The PCA was performed in cryoSPARC v3 (44) using 3D variability script with a set of 1,401,983 particle images, which were not subjected to prior 3D classifications or 3D refinements. We calculated three eigenvectors of the 3D covariance (Fig. S13). The molecular model of Stx2a–P-stalk was docked into the output maps representing the first eigenvector of the 3D covariance. The map models were then inspected in COOT 0.9 and refined in Phenix 1.18 as described in the previous section concerned with cryo-EM structure determination, model building, and refinements.

The structural model of the pentameric P-stalk was built with ColabFold:AlphaFold2 (Deep Mind) using MMseq2 (Max-Planck Institute for Biophysical Chemistry) (53). Five models were generated with three recycles. Disorder predictions were performed with PrDOS (54).

## Data availability

Atomic coordinates of the structure of Stx2a bound to the ribosomal P-stalk were deposited to the PDB under an accession number 7U6V. Cryo-EM maps were deposited to the Electron Microscopy Data Bank under accession numbers: EMD-26381 (Stx2a–ribosomal P-stalk complex) and EMD-26380 (*S. cerevisiae* 80S ribosome).

## Supporting information

Supporting information include 1 table, 1 movie, 13 figures, experimental procedures and results, which can be found at the *Journal of Biological Chemistry* website.

## Conflict of interest

The authors declare that they have no conflicts of interest with the contents of this article.
