# Supplementary material for: Cryo-EM structure of Shiga toxin 2 in complex with the native ribosomal P-stalk reveals residues involved in the binding interaction
Source: J Biol Chem. 2022 Dec 15;299(1):102795. doi: 10.1016/j.jbc.2022.102795 (PMC9823235; doi:10.1016/j.jbc.2022.102795)
Supplement: Supporting information [file mmc2.pdf]

Cryo-EM structure of Shiga toxin 2 in complex with the native ribosomal P-stalk

Arkadiusz W. Kulczyk, Carlos Oscar S. Sorzano, Przemysław Grela, Marek Tchórzewski, Nilgun E. Tumer and Xiao-Ping Li

List of included materials:

Table S1. Cryo-EM data collection and processing statistics.

Movie S1. Conformational changes in the structure of the Stx2a-P-stalk complex.

Fig. S1. SPR studies of the interaction between Stx2a and the native ribosomal P-stalk.

Fig. S2. Negative-stain EM analysis of the Stx2a-P-stalk complex.

Fig. S3. The SPA workflow employed for cryo-EM structure calculations.

Fig. S4. Cryo-EM processing workflow employed for Stx2a-P-stalk structure calculations.

Fig. S5. Structure calculations of the Stx2a-P-stalk complex.

Fig. S6. Resolution estimates for the Stx2a-P-stalk structure.

Fig. S7. Validation of the Stx2a-P-stalk model.

Fig. S8. Structural features of the Stx2a-P-stalk complex.

Fig. S9. Superposition of cryo-EM and crystal structures of Stx2a.

Fig. S10. Cryo-EM analysis of Stx2a in complex with *S. cerevisiae* ribosome.

Fig. S11. AlphaFold2 structural models of the pentameric P-stalk.

Fig. S12. Superposition of an ensemble of NMR structures of P1A and P2B and AlphaFold2 models.

Fig. S13. Principal Components Analysis of Stx2a-P-stalk dynamics.

Cryo-EM analysis of Stx2a-ribosome binding, and determination of *S. cerevisiae* ribosome structure.

Supporting experimental procedures.

Supporting results.

Data collection	
Electron microscope	Titan Krios
Camera	K3
Magnification	105,000x
Voltege (kV)	300
Dose rate (e-/Å2/s)	29.41
Acumulated dose (e-/Å2)	58.82
Exposure time (s)	2
Defocus range (µm)	0.8-2.5
Pixel size (Å)	0.4124
Movie frames	40
Total movies (no.)	35,052
Initial particle images (no.)	1,401,983
**Map**	
EMDB accession code	EMD-26381
Resolution (Å)	4.1
Local resolution (Å)	3.5-7.5
FSC threshold	0.143
Software	RELION 3, cryoSPARC v3, Scipion 3
Symmetry	C1
No. of particles	112,924
**Model**	
PDB accession code	7U6V
**Model building**	
Software	Coot 0.9
**Refinement**	
Software	Phenix 1.18
**Model composition**	
Chains	8
Residues	637
**Validation**	
MolProbity score	1.2
Clashscore	13.54
R.m.s. deviations	
Bond lengths (Å)	0.002
Bond angles (0)	0.439
**Ramachandran**	
Favored (%)	97.9
Allowed (%)	2.1
Outliers (%)	0

Table 1. **Cryo-EM data collection and processing statistics.**

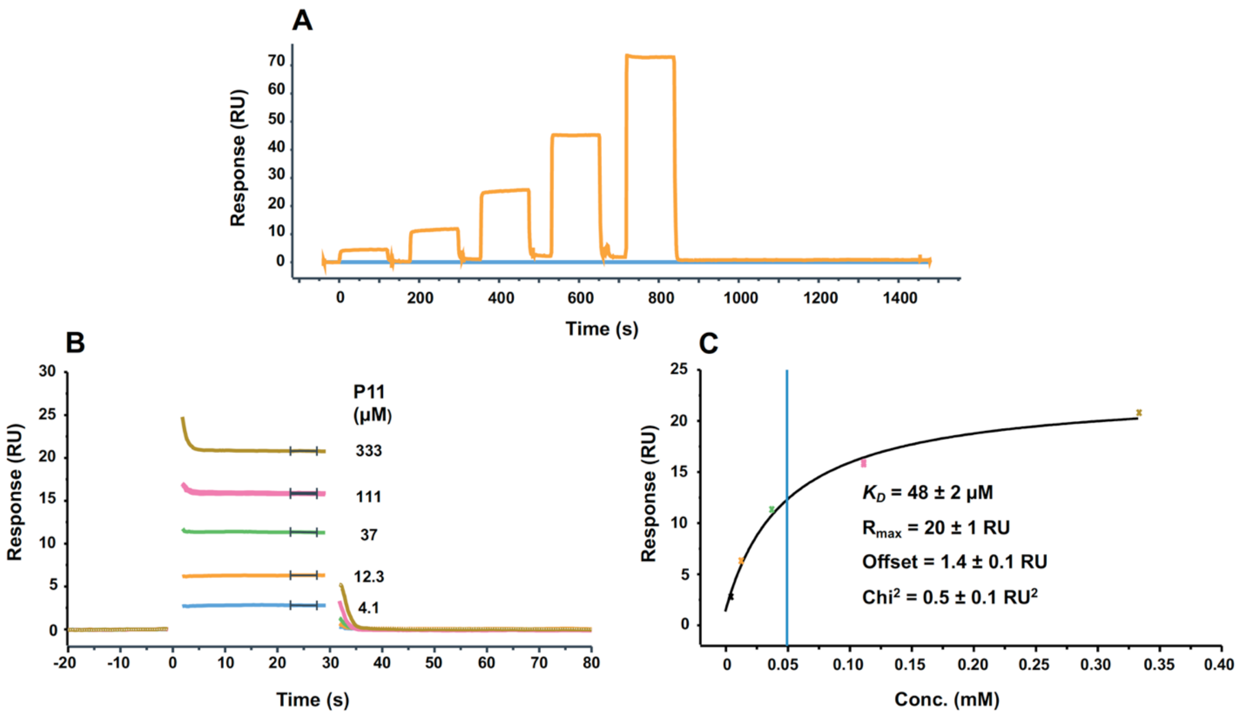

FIGURE S1**. SPR studies of the interaction between Stx2a and the native ribosomal P-stalk or P11.** A, the Stx2a-P-stalk interaction was measured with Biacore 8K$^+$ using the single-cycle kinetics method. Stx2a was immobilized on Fc2 of a CM5 chip at 800-820 RU using amine coupling. Fc1 was activated and blocked using the same reagent. The yeast P-stalk pentamer was flown over the surface of the chip at the following concentrations: 49.4 nM, 148.1 nM, 444.4 nM, 1.3 µM and 4 µM. The running buffer contained 10 mM HEPES pH 7.4, 150 mM NaCl, 3 mM EDTA, 0.05% Surfactant P20 and 10 mM $MgCl_2$. The figure shows one of the two replicates. B, interaction sensorgrams measured for Stx2a and P11. Stx2a holotoxin was immobilized on Fc2 of a CM5 chip (1400-1600 RU) using amine coupling in 4 different channels. P11 was flown over the surface of the chip at the following concentrations: 4.1 µM, 12.3 µM, 37 µM, 111 µM, and 333 µM. The running buffer contained: 10 mM HEPES pH 7.4, 150 mM NaCl, 3 mM EDTA, 0.05% P20, and 10 mM $MgCl_2$. C, the binding affinity (K_D) of Stx2a and P11 was calculated by fitting results at steady state. The blue line denotes the K_D. The fitting results are shown as an average of 4 replicates from 4 channels.

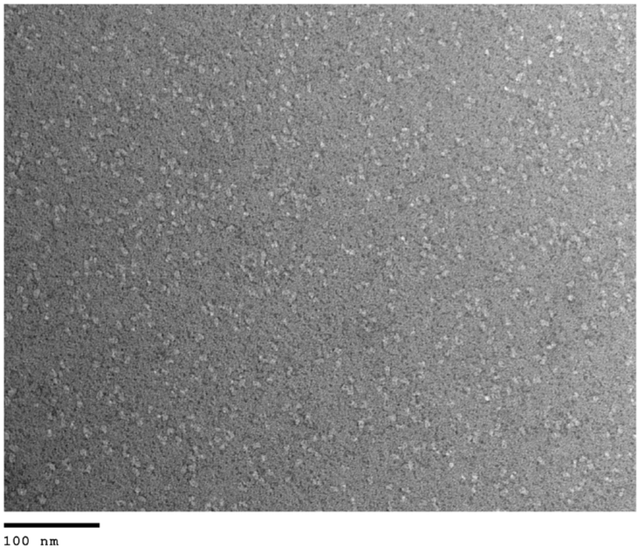

FIGURE S2. **Negative-stain EM analysis of Stx2a-P-stalk complex.** The micrograph shows approximately 500-800 particles, which are well-dispersed across the field of view. The samples were stained with 2% (w/v) uranyl formate for 3 min at room temperature. The micrograph was acquired using Philips CM-12 electron microscope operating at 80 kV acceleration voltage at a magnification of 60,000 times corresponding to the pixel size of 1.95 Å/px.

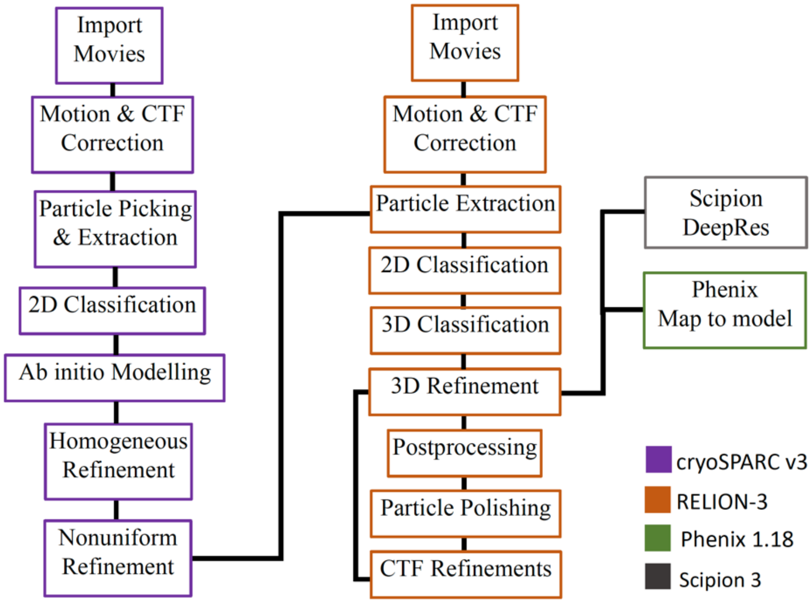

FIGURE S3. **The SPA workflow employed for cryo-EM structure calculations.** We employed cryoSPARC v3, RELION-3, Phenix 1.18 and Scipion 3 for image processing and structure calculations of the Stx2a-P-stalk complex. Steps completed in cryoSPARC v3, RELION-3, Phenix 1.18, and Scipion 3 are denoted in purple, orange, green, and gray boxes, respectively.

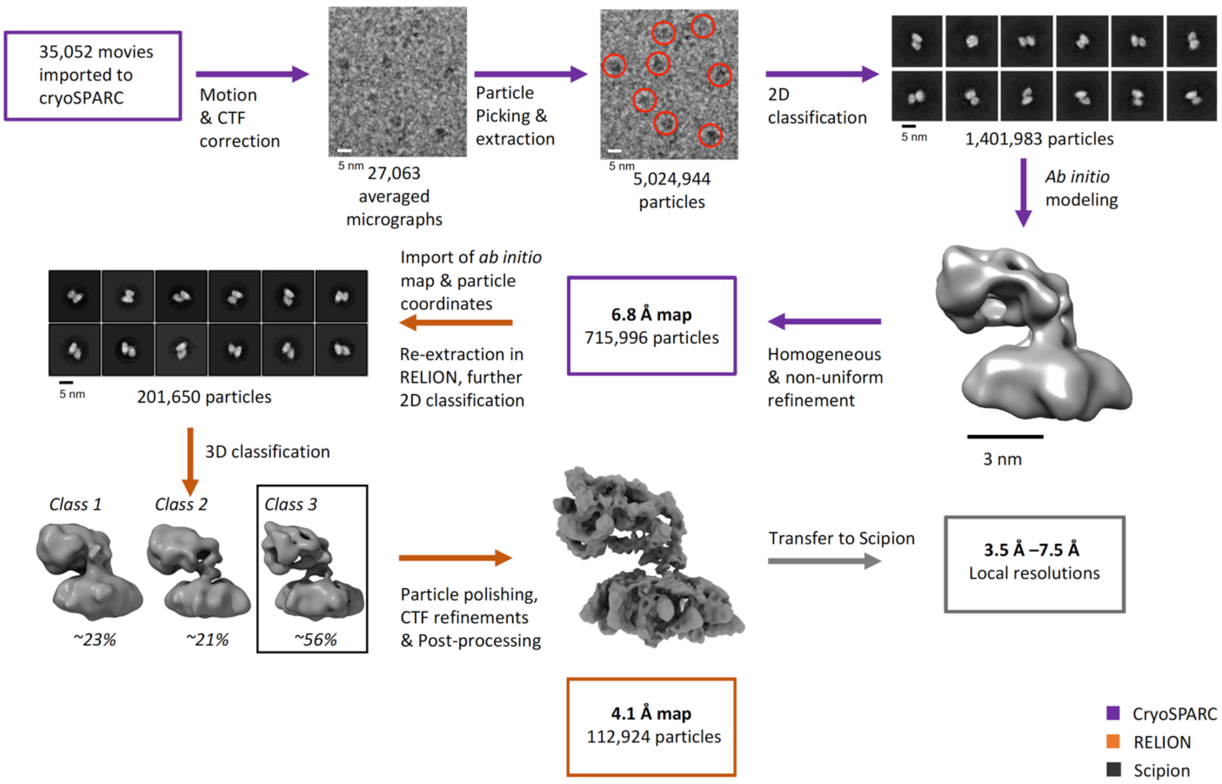

FIGURE S4. **Cryo-EM processing workflow employed for Stx2a-P-stalk structure calculations.** Steps completed in cryoSPARC v3, RELION-3 and Scipion 3 are denoted in purple, orange and gray, respectively. An averaged micrograph, representing the first step of processing, displays a uniform distribution of particles throughout the image. An identical averaged micrograph is displayed again to illustrate the robustness of the automated particle picking from the preprocessed images. The selected particles are indicated by red circles in the image. Please see Experimental Procedures and Fig. S3 for more details.

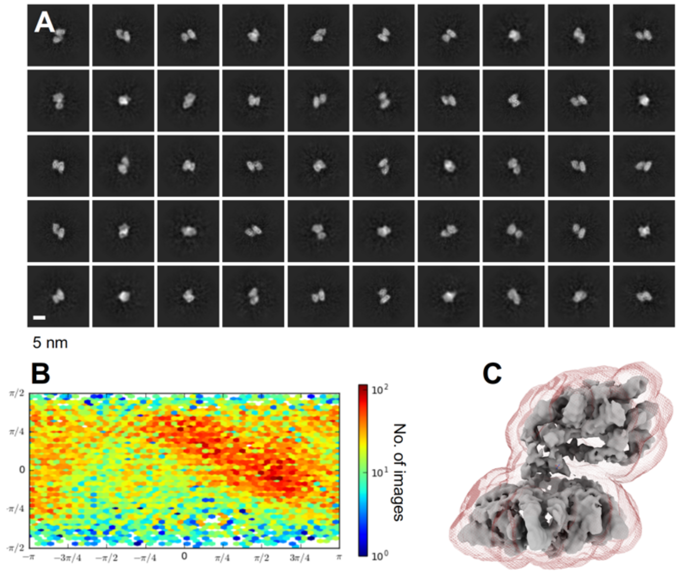

FIGURE S5. **Structure calculations of the Stx2a-P-stalk complex.** A, representative 2D class averages calculated for Stx2a-P-stalk in cryoSPARC reveal multiple views of the complex. B, the angular distribution heatmap confirms the complete coverage of the angular space. C, the mask applied during refinements in RELION-3 was not too tight, and it encompassed all parts of the 3D map.

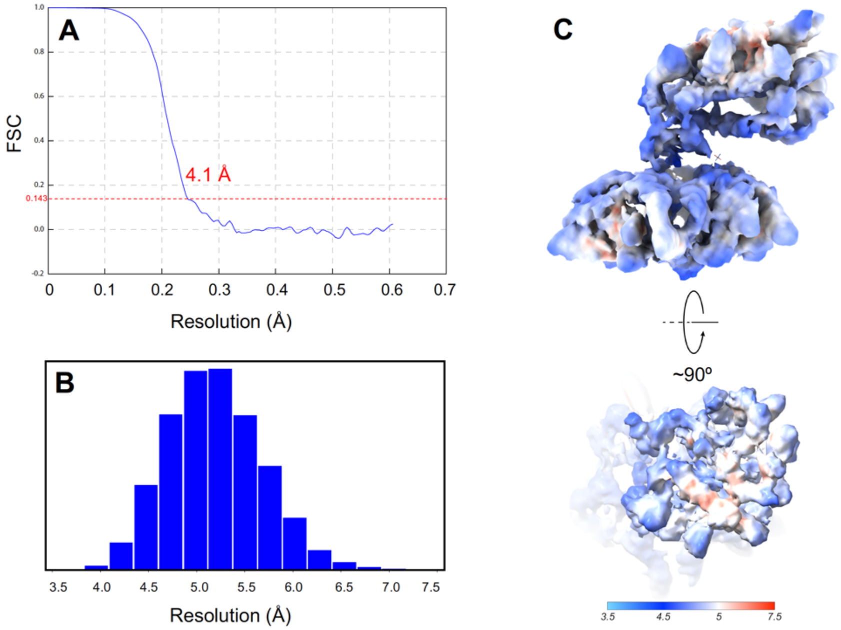

FIGURE S6. **Resolution estimates for the Stx2a-P-stalk structure.** A, resolution of the Stx2a-P-stalk structure was calculated using the “gold standard” Fourier Shell Correlation (FSC) method according to the 0.143 criterion in RELION-3. B, local resolution histogram calculated using the program DeepRes in Scipion 3. C, local resolution values mapped onto the surface of the Stx2a-P-stalk structure.

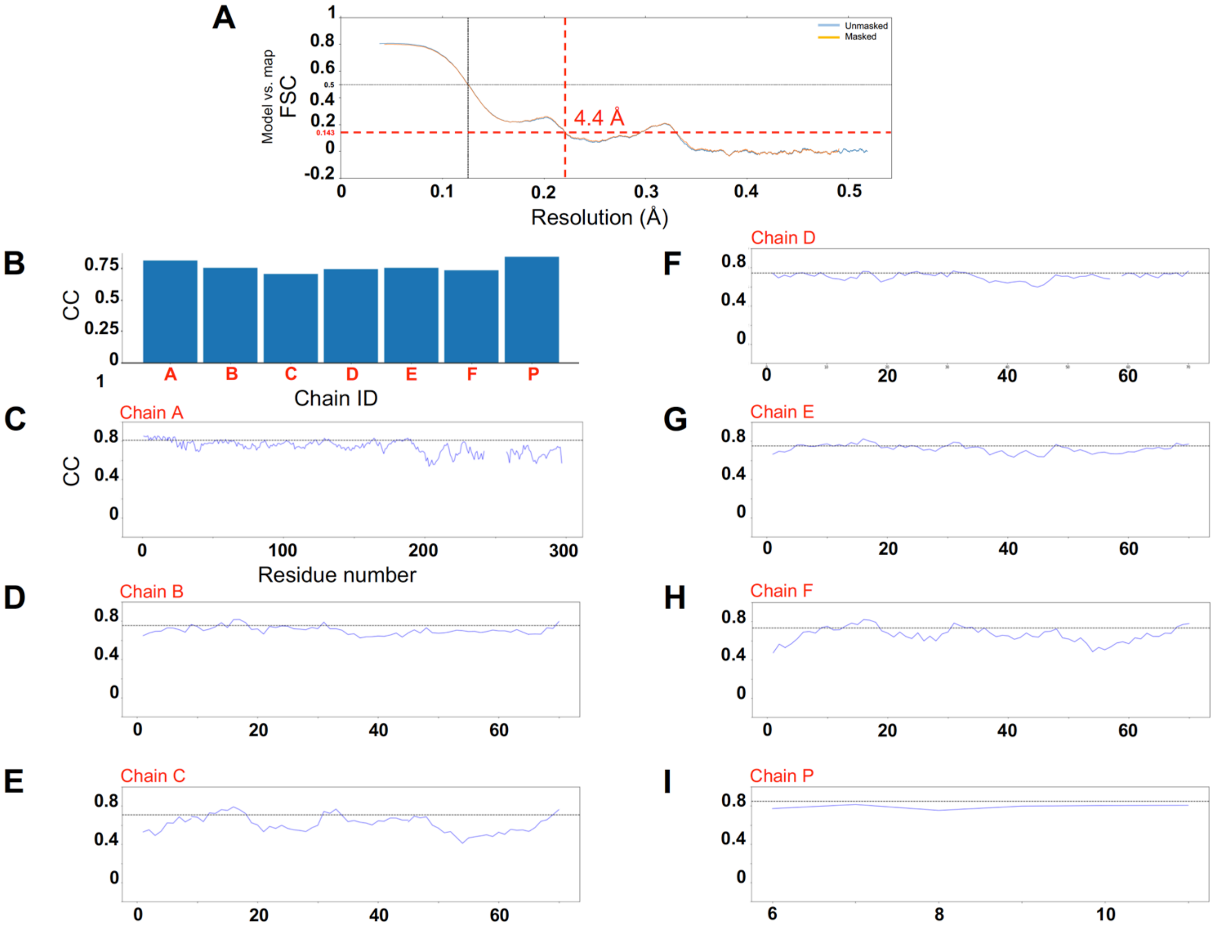

FIGURE S7. **Validation of the Stx2a-P-stalk model.** A, the output from phenix.mtriage. The graph shows FSC curves representing the model vs. map cross-correlation with and without masking. The model displays a good agreement with the map, with the FSC at 0.143 of 4.4 Å consistent with the reported resolution of the map. Please see Figure S6 for more details. B, the output from phenix.validation_cryoem showing cross-correlation of individual protein chains and the map. C-I, the output from phenix.validation_cryoem displaying cross-correlation coefficients calculated for the model vs. map per residue for each individual chain.

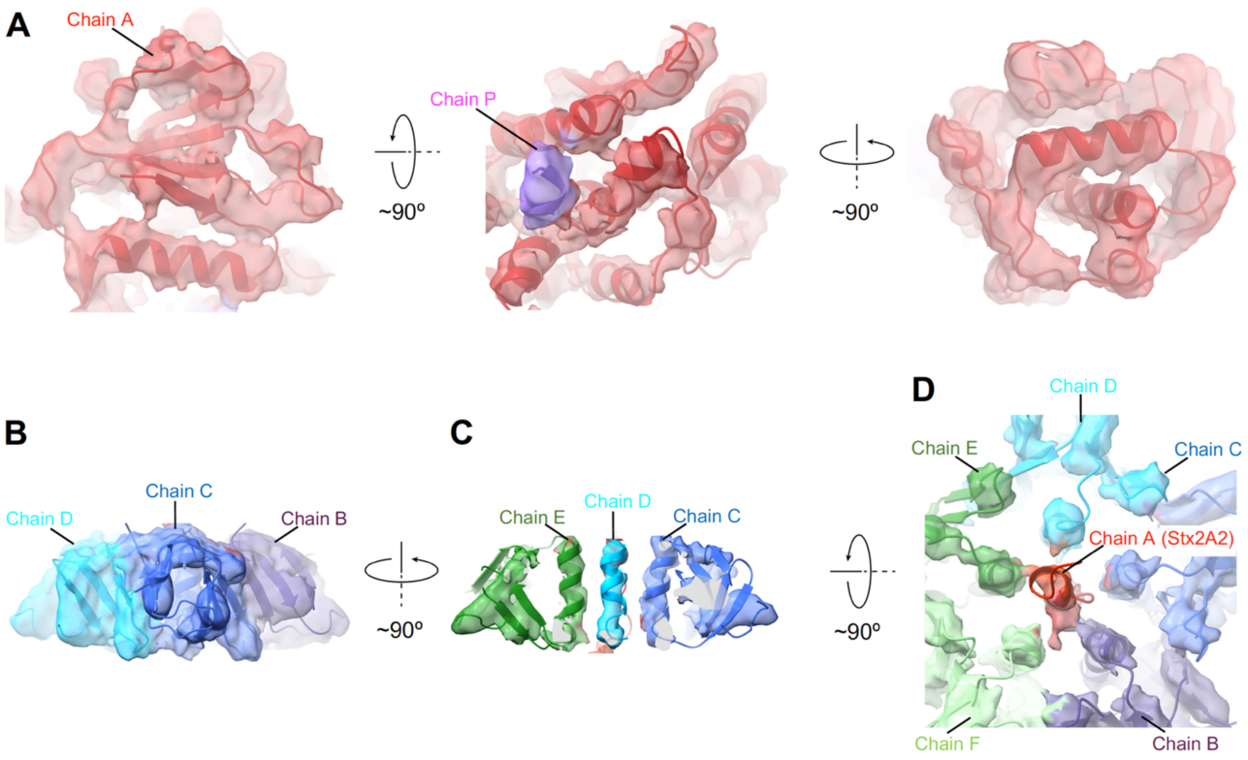

FIGURE S8. **Structural features of the Stx2a-P-stalk complex.** A, different views of the chain A (Stx2A1) and the chain P (CTD hexapeptide). B, a side view of the surface of Stx2B containing chains B, C, and D. C, a vertical slice through the map of Stx2B displaying densities representing chains C, D, and E. D, a horizontal slice-through Stx2B showing densities representing chains B, C, D, E, F, and Stx2A2 i.e. a part of the chain A shown in red, which inserts into a channel formed by a pentameric ring of Stx2B.

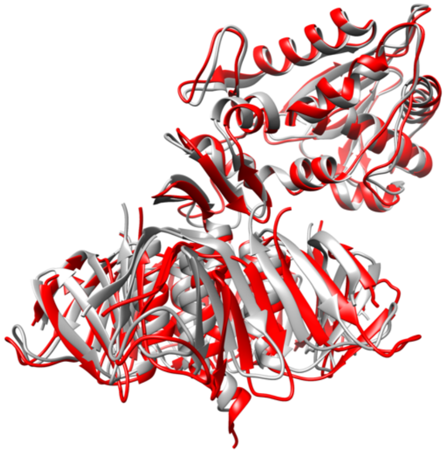

FIGURE S9. **Superposition of cryo-EM and crystal structures of Stx2a.** The cryo-EM structures of Stx2a-P11 show good overall agreement with a structure of Stx2a-P11 determined by X-ray crystallography (PDB IDs: 6X6H) with backbone RMSD of 1.84 Å (Stx2A) and 0.94 Å – 1.94 Å (individual Stx2B subunits). However, the analysis of cryo-EM data reveals an extensive dynamical behavior of Stx2a-P-stalk (Movie S1).

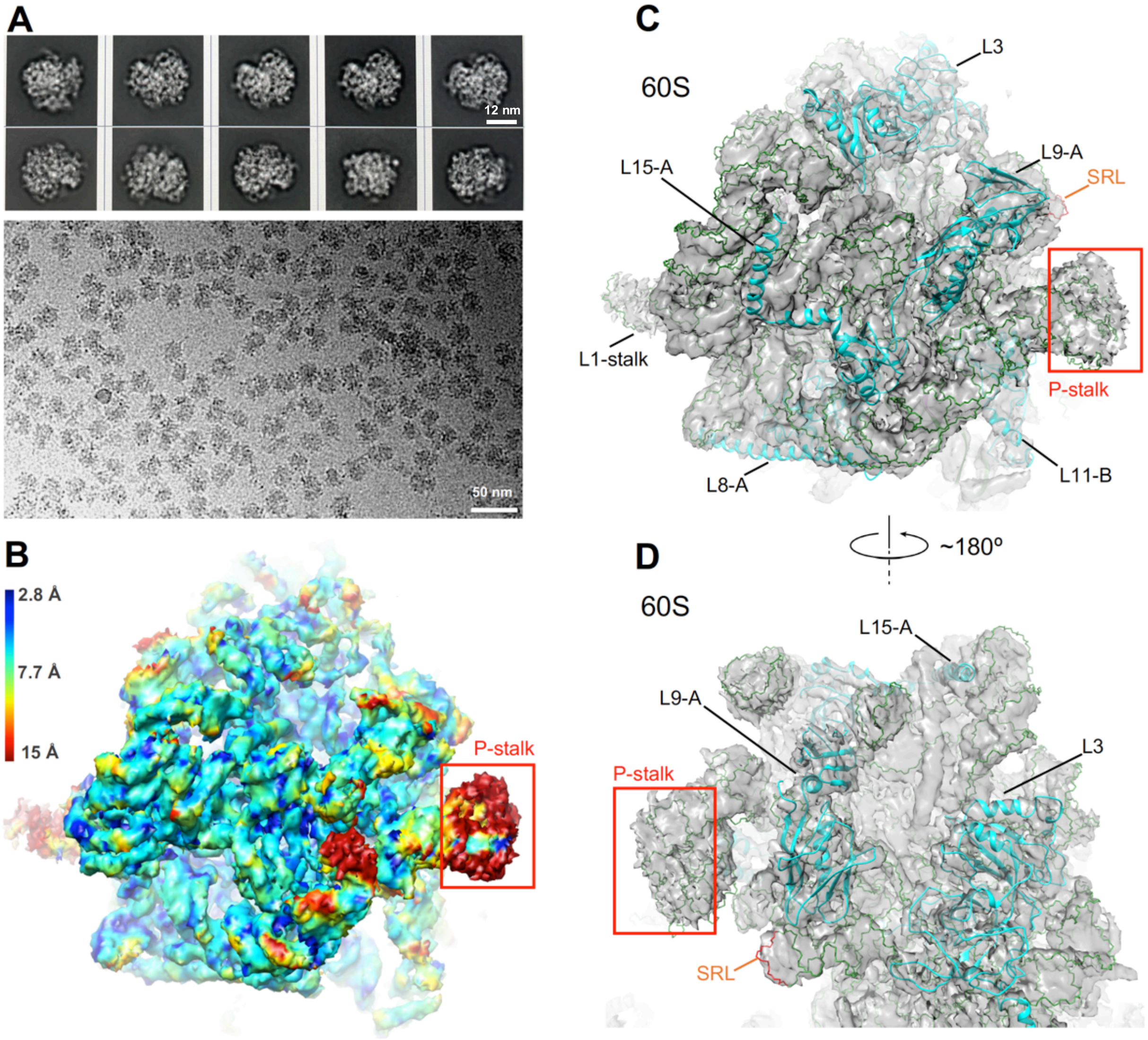

FIGURE S10. **Cryo-EM structure of the *S. cerevisiae* ribosome.** A, representative micrograph and 2D class averages. The micrograph shows approximately 500 particles dispersed across a field of view. Representative 2D class averages reveal well-defined densities representing 40S and 60S ribosomal subunits. B, cryo-EM structure of the *S. cerevisiae* ribosome. The picture shows local resolution estimates mapped onto the surface of 60S subunit. Resolution estimates at the base of the ribosomal P-stalk (~15 Å, red box) indicate flexibility of the P-stalk in solution. C and D, structural features of the cryo-EM map. 60S rRNA is shown in gray and ribosomal proteins are displayed in cyan. The base of the ribosomal P-stalk and the sarcin-ricin loop (SRL) are indicated in orange. Details of the cryo-EM structure determination of the *S. cerevisiae* ribosome are described in the Supporting Experimental Procedures and in the Supporting Results on pages S13-S14.

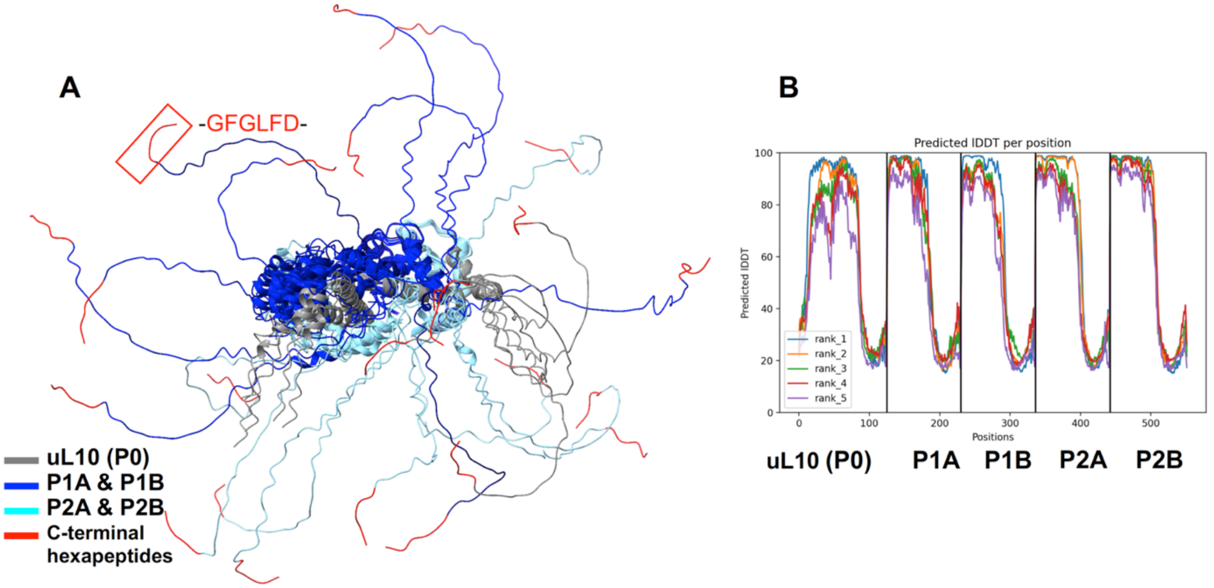

FIGURE S11. **AlphaFold2 structural models of the pentameric P-stalk.** A, five predicted P-stalk models (ranks 1-5). The uL (P0), P1A/P1B and P2A/P2B are displayed in gray, dark blue and light blue, respectively. The C-terminal hexapeptides (GFGLFD) are shown in red. One of the hexapeptides is highlighted in the red box. B, The Local Distance Difference Test (lDDT) values calculated for each AlphaFold2 model and rank. The “rank 1” model of the P-stalk pentamer calculated with a total of 552 amino acids and containing uL10 (P0), P1A, P1B, P2A and P2B subunits is also displayed in Figure 6A.

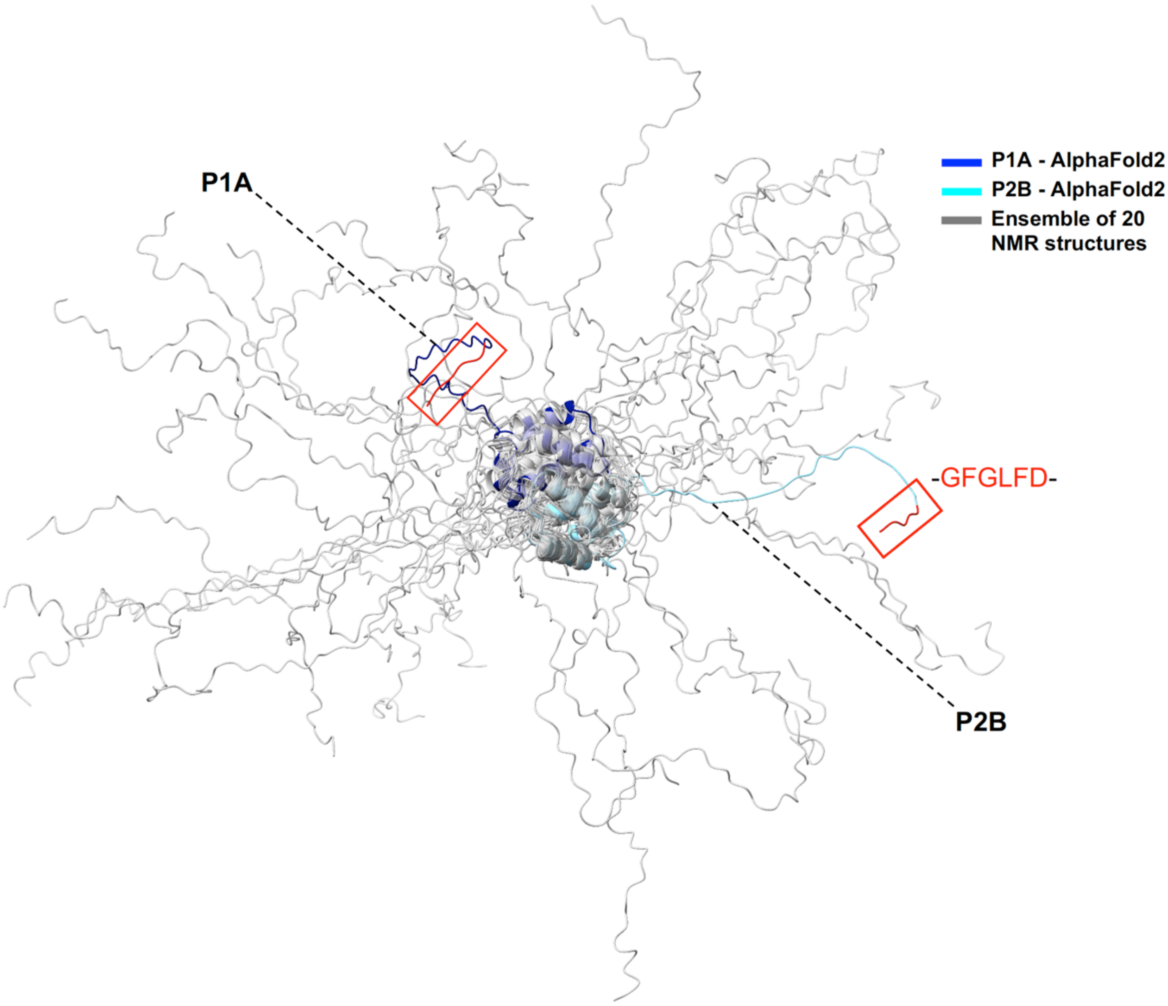

FIGURE S12. **Superposition of an ensemble of NMR structures of P1A and P2B and AlphaFold2 models.** The NMR structure of the P1A-P2B dimer (PDB ID: 4BEH) was superimposed with a part of the AlphaFold2 model of the P-stalk pentamer representing the P1A and P2B chains. The complete Alphafold2 models are presented in Figure 6A and in Figure S11A. The backbone RMSDs are 1.2 Å (P1A) and 0.9 Å (P2B) across the 4-helix bundles forming the N-terminal domains of both proteins. One of the C-terminal CTD hexapeptides (GFGLFD) is highlighted in the red box.

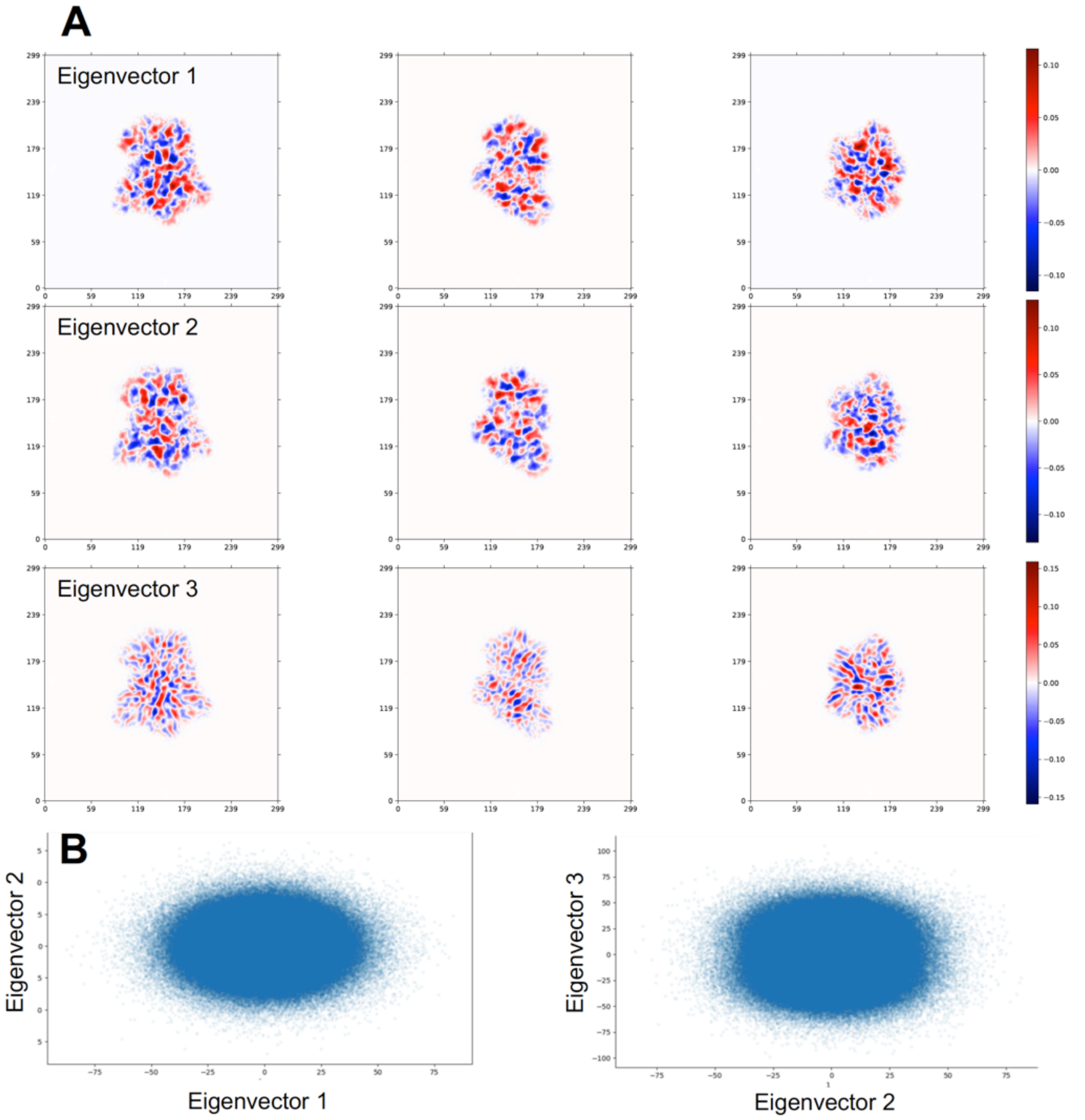

FIGURE S13. **Principal Components Analysis of the Stx2a-P-stalk dynamics.** A, three eigenvectors representing variability in the data-set of 1,401,983 particle images. The variability is characterized by the range of positive (red) and negative (blue) values at each voxel of the volume. Three different projections of each eigenvector volume are shown. B, reaction coordinates calculated for the set of 1,401,983 particle images. The distribution of reaction coordinates across particles in the dataset provides information about the variability landscape of the complex by highlighting the populations of particles adopting a specific position in the conformational space spanned by the eigenvectors. These plots illustrate the mobility within the Stx2a-P-stalk complex. The continuous distributions confirm the flexing motion of the molecule, rather than the presence of discrete conformations.

Cryo-EM analysis of Stx2a-ribosome binding, and determination of *S. cerevisiae* ribosome structure.

SUPPORTING EXPERIMENTAL PROCEDURES

Purification of S. cerevisiae 80S ribosomes

Purification of *S. cerevisiae* 80S ribosomes was performed according to the protocol described earlier (27).

Negative-stain analysis

The samples were first screened by negative-stain EM. Stx2a and *S. cerevisiae* ribosomes were mixed in a ratio ranging from 1:1 to 1:30 (ribosome:Stx2) at the concentration in the range 0.3-1.3 mg/ml in 50 mM HEPES buffer pH 7.5 containing 50 mM NH_4Cl and 5 mM $Mg(CH_3COO)_2$, and incubated for 10 min at room temperature. The 3 μl aliquots of samples were applied to carbon-coated 300-mesh copper grids, which were previously rendered hydrophilic with PELCO easiGlow glow discharger. Grids were stained with 2% (w/v) uranyl formate for 3 min at room temperature. After staining, grids were air-dried and inserted into the microscope. Images were recorded using Philips CM-12 electron microscope operating at 80 kV acceleration voltage at a magnification of 75,000 times corresponding to the pixel size of 2.44 Å/px.

Cryo-EM sample preparation, screening and data collection

Samples of the Stx2a-ribosome complexes were prepared by mixing individual proteins in a buffer described above. Samples used for final data collections consisted of 1.29 mg/ml complex formed after mixing ribosome:Stx2a in 1:30 stoichiometry. This concentration is ~45 times higher than the dissociation constant for complex formation. Samples were adsorbed onto freshly glow-discharged 300 mesh gold Quantifoil R2/1 grids with PELCO easiGlow glow discharger and flash frozen in liquid ethane using either Vitrobot Mark IV dual-blotting plunger or Leica GP Climate controlled sample plunger with a controlled temperature and humidity. The grids were screened with a 200kV Thermo Fisher Scientific Talos Arctica electron microscope equipped with a Gatan BioQuatum energy filter and Gatan K2 Summit direct electron detector at the Center for Integrative Proteomics Research at Rutgers University using the software SerialEM or EPU for automated data collection. We used the following acquisition parameters for the final data collection: dose rate of 6.45 e^-/px/s in a counting mode, magnification of 130,000 times corresponding to the pixel size of 1.037 Å/px with a defocus range -0.5 to -2.5 μm. We collected 20 frames with 200 ms per frame exposure, a total exposure of 4 s and an accumulated dose of 25.8 e^-.

Structure determination

Structures of the ribosome-Stx2a complex was calculated using Scipion 3. Three thousand two hundred and eleven imported movies were motion-corrected with UNBLUR and CTF-corrected using CTFFIND4. Pre-processed images were used for particle picking using XMIPP3 – manual-picking and XMIPP3 – auto-picking scripts. A set of 123,952 particle images was then extracted and screened in XMIPP3. After screening, 117,042 particles were subjected to 2D classification and averaging in RELION-3 and XMIPP3 – cl2d. During 2D analysis artifacts and particles not converging to stable classes were removed from the data set resulting with a set of 116,850 particles. The initial volume was created using XMIPP3 – reconstruct significant. The resultant volume and output particles obtained following 2D classification and averaging were used for 3D classification in RELION-3 with 5 classes. One of the classes containing 63,689 particles clearly displayed well-defined densities representing small and large ribosomal subunits, whereas other classes contained dissociated ribosomes. This class was used for further refinement in RELION-3 – 3D auto-refine producing a 6.4 Å structure of the ribosome. This structure was used as an input volume for 1 round of global and 5 rounds of local refinements with XMIPP-3 – highres. By applying multiple rounds of refinements with gradually decreasing target resolution, we were able to refine the ribosome structure with local resolution reaching 2.8 Å according to a gold standard FSC 0.143 criterion. Local resolution of the resultant structure was estimated according to the gold standard Fourier Shell Correlation (FSC) 0.143 criterion using the program XMIPP3 – local

MonoRes. In an attempt to visualize the density representing Stx2a bound to the P-stalk, we carried out focused classification with signal subtraction with RELION-3 – subtract projection and XMIPP3 – reconstruct fourier, using a mask created in UCSF Chimera. This mask excluded all parts of the ribosome, except for the region adjacent to the ribosomal P-stalk.

SUPPORTING RESULTS

Cryo-EM analysis of the Stx2a-ribosome binding

To examine if the binding of Stx2a to the entire ribosome, including the P-stalk, would stabilize the CTD-Stx2a such that a fragment of the P-stalk reaching beyond the C-terminal six amino-acids can be visualized, we attempted to determine a structure of Stx2a complexed with the 3 MDa *S. cerevisiae* ribosome. We collected and processed cryo-EM images (Fig. S10A) and determined a structure of the 80S ribosome but we were not able to visualize Stx2a binding. The ribosome structure revealed an extended density including a part of the ribosomal P-stalk, most likely a fragment of the uL10 protein (Fig. S10B-D). We were not able to visualize densities representing C-termini of P1/P2 proteins. Local resolution in the extended part of the P-stalk structure is ~15 Å (Fig. S10B), indicating high mobility of the stalk protuberance composed of P1/P2 dimers. In an effort to visualize the density representing Stx2a bound to the ribosome, we carried out masked classification and focused classification with signal subtraction in RELION-3 and Scipion 3 but this approach was not successful. The results confirm that the interaction of the ribosome and Stx2a is dynamic in nature, the observation consistent with previously published structural and biochemical data.
